# Interaction between the tRNA-Binding and C-Terminal Domains of Yeast Gcn2 Regulates Kinase Activity In Vivo

**DOI:** 10.1371/journal.pgen.1004991

**Published:** 2015-02-19

**Authors:** Sebastien Lageix, Jinwei Zhang, Stefan Rothenburg, Alan G. Hinnebusch

**Affiliations:** 1 Laboratory of Gene Regulation and Development, Eunice K. Shriver National Institute of Child Health and Human Development, National Institutes of Health, Bethesda, Maryland, United States of America; 2 National Heart, Lung and Blood Institute, National Institutes of Health, Bethesda, Maryland, United States of America; 3 Division of Biology, Kansas State University, Manhattan, Kansas, United States of America; faculty of Life Sciences, University of Manchester, UK, UNITED KINGDOM

## Abstract

The stress-activated protein kinase Gcn2 regulates protein synthesis by phosphorylation of translation initiation factor eIF2α. Gcn2 is activated in amino acid-deprived cells by binding of uncharged tRNA to the regulatory domain related to histidyl-tRNA synthetase, but the molecular mechanism of activation is unclear. We used a genetic approach to identify a key regulatory surface in Gcn2 that is proximal to the predicted active site of the HisRS domain and likely remodeled by tRNA binding. Mutations leading to amino acid substitutions on this surface were identified that activate Gcn2 at low levels of tRNA binding (Gcd^-^ phenotype), while other substitutions block kinase activation (Gcn^-^ phenotype), in some cases without altering tRNA binding by Gcn2 in vitro. Remarkably, the Gcn^-^ substitutions increase affinity of the HisRS domain for the C-terminal domain (CTD), previously implicated as a kinase autoinhibitory segment, in a manner dampened by HisRS domain Gcd^-^ substitutions and by amino acid starvation in vivo. Moreover, tRNA specifically antagonizes HisRS/CTD association in vitro. These findings support a model wherein HisRS-CTD interaction facilitates the autoinhibitory function of the CTD in nonstarvation conditions, with tRNA binding eliciting kinase activation by weakening HisRS-CTD association with attendant disruption of the autoinhibitory KD-CTD interaction.

## Author Summary

The survival of all living organisms depends on their capacity to adapt their gene expression program to variations in the environment. When subjected to various stresses, eukaryotic cells modulate general and gene-specific protein synthesis by phosphorylating the α-subunit of eukaryotic translation initiation factor 2 (eIF2α). The yeast *Saccharomyces cerevisiae* has a single eIF2α kinase, Gcn2, activated by uncharged tRNAs that accumulate in amino acid starved cells, which bind to a regulatory domain homologous to histidyl-tRNA synthetase (HisRS). Gcn2 also contains a C-terminal domain implicated in autoinhibition of Gcn2. Our findings identify a direct interaction between the CTD and a novel regulatory surface in the HisRS domain that is required for inhibition of Gcn2 function in non-starved cells, which is down-regulated by uncharged tRNA. The results further suggest that tRNA binding to the pseudo-active site in the HisRS domain remodels its proximal CTD-binding surface to weaken HisRS/CTD interaction and thereby release the autoinhibitory function of the CTD to activate kinase function. This study provides new molecular insights into how tRNA binding can modulate regulatory interactions among the HisRS, CTD, and kinase domains of Gcn2 to elicit kinase activation.

## Introduction

Eukaryotic cells harbor stress-activated protein kinases that allow cells to reduce bulk protein synthesis while simultaneously activating the transcription of genes encoding stress management proteins. The target of these kinases is Ser-51 of the α-subunit of translation initiation factor 2 (eIF2α). Phosphorylation of eIF2α reduces the function of eIF2 in recruiting methionyl initiator tRNA to the 40S ribosomal subunit by impairing the recycling of eIF2-GDP to eIF2-GTP by guanine exchange factor eIF2B and thereby reducing the cellular concentration of eIF2·GTP·Met-tRNA_i_^Met^ ternary complexes. The inhibition of ternary complex assembly diminishes the rate of general translation but enables translation preinitiation complexes to bypass multiple “decoy” AUG start codons in the mRNA leader of *GCN4* mRNA and translate the coding sequences for Gcn4, a transcriptional activator of amino acid and vitamin biosynthetic genes in budding yeast (reviewed in [1]). A similar mechanism up-regulates translation of mammalian *ATF4* and *ATF5* mRNAs when eIF2α is phosphorylated by Gcn2 or one of the other mammalian eIF2α kinases PKR, PERK and HRI [2] [3]. PKR is a key component of the innate immune response, PERK is crucial for responding to ER stress, and HRI couples globin synthesis to heme availability in reticulocytes [4]. Interestingly, rodent Gcn2 mediates the animal’s aversion to amino acid-deficient diets [5], dampens protein synthesis in muscle during leucine starvation [6], and functions in lipid homeostasis [7] and in learning and memory formation [8]. Mammalian Gcn2 has also been implicated in tumor cell survival, innate and T-cell mediated immune responses, and DNA repair (reviewed in [9]); and recently mutations in human Gcn2 were linked to pulmonary hypertension [10]. Hence, elucidating the molecular mechanism of Gcn2 regulation is of importance to multiple aspects of human development and physiology.

**Fig 1:**
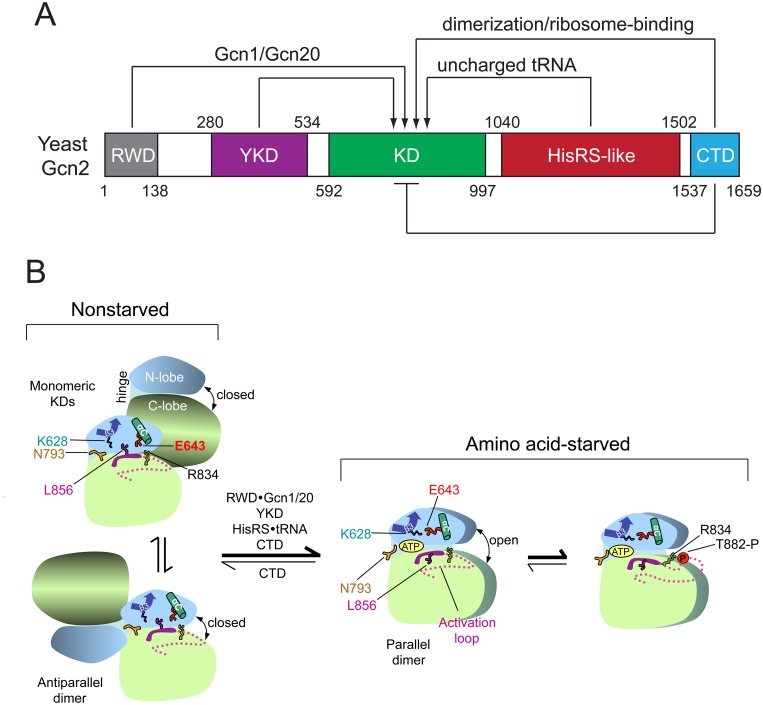
Summary of domain interactions in Gcn2 that couple binding of uncharged tRNA to activation of kinase function in starved cells. **(A)** Schematic diagram of the domain structure of yeast Gcn2 and interdomain interactions controlling kinase activity. The amino acid coordinates of the RWD/GI domain (pdb 2yz0), pseudokinase domain (YKD) [21], kinase domain (KD) [12], HisRS-like domain [14], and CTD [26] are derived from crystal structures (RWD, KD, CTD) or multiple sequence alignments (YKD and HisRS-like) of the respective domains. Stimulatory interactions are depicted with arrows, and involve domain interactions with Gcn1/Gcn20 (RWD) or uncharged tRNA (HisRS-like); or dimerization and ribosome-binding activities (CTD). The CTD also mediates autoinhibition of kinase activity, depicted with a bar. **(B)** Schematic summary of conformational rearrangements in the KD evoked by uncharged tRNA binding to the HisRS domain in amino acid-starved cells. The mode of dimerization and disposition of helix αC, β strand-3 (β3), and the activation loop, as well as key residues in the KD controlling ATP binding and catalysis (Lys-628, Asn-793, Glu-643, Arg-834, and Leu-856) are depicted for the inactive and active states of the Gcn2 KD that prevail in nonstarved or amino acid-starved cells, respectively. It is assumed that, in both states, Gcn2 dimerizes through self-interaction of the CTD and the schematic depicts only the disposition of the KDs within the full-length dimer. At low levels of uncharged tRNA in nonstarved cells (*left*), the KD exists in an equilibrium between two inactive conformations, with monomeric KDs (*upper*) or the KDs in an antiparallel dimer (*lower*). In both, ATP binding is hindered by a closed conformation of the N- and C-lobes and by Gln-793 (N793), which forms a flap over the ATP-binding pocket; and catalysis is blocked by hinge rigidity and rotation of αC to a nonproductive orientation that is stabilized by L856 and the inhibitory E643-R834 salt bridge. CTD/KD domain interaction also promotes this inactive conformation by an unknown mechanism. In amino acid-starved cells, binding of uncharged tRNA to the HisRS domain, along with stimulatory contributions of the RWD domain (engaged with Gcn1/Gcn20), the YKD domain, and the CTD, evokes a parallel mode of KD dimerization with αC properly oriented to form the stimulatory K628-E643 salt-bridge, and with ATP binding enhanced by a reduction in hinge rigidity that facilitates an open conformation of the N- and C-lobes and displaces the inhibitory N793 flap. Autophosphorylation of the activation loop ensues to produce a fully functional kinase locked into the active conformation. (See text for additional details; modified from Garriz et al [13].)

Because eIF2α kinases act by inhibiting translation, their functions must be tightly regulated to limit eIF2α phosphorylation to the appropriate stress conditions. The Gcn2 kinase domain (KD) is intrinsically inert and depends on interactions with four other domains within Gcn2 to achieve an active conformation (Fig. 1A) [11]. Latency of Gcn2 KD activity depends on a rigid hinge connecting the N- and C-lobes of the KD, promoting a partially closed active site cleft and occluded ATP-binding pocket, and also on a non-productive orientation of helix αC in the N-lobe that blocks the proper positioning of ATP phosphates for catalysis (Fig. 1B) [12,13]. Binding of uncharged tRNA to the region immediately C-terminal to the KD, related in sequence to histidyl-tRNA synthetase (HisRS) is required to activate Gcn2 in amino acid-starved cells (Fig. 1A) [14,15,16,17]. Authentic HisRS is the enzyme that aminoacylates tRNA^His^ for protein synthesis. Consistent with the fact that Gcn2 is activated by starvation for any amino acid [15], the Gcn2 HisRS-related domain (henceforth, just HisRS domain) is not specific for binding histidyl tRNA [17]. An N-terminal segment in the HisRS domain that interacts with a portion of the KD containing the hinge is required for kinase activation [18], suggesting that tRNA binding might alter the HisRS-KD interface to evoke an active conformation of the KD. A pseudokinase domain (YKD), incapable of binding ATP or Mg^+2^ in vitro [19], is located just N-terminal to the KD and is also required for kinase activation (Fig. 1A) [16,20]. Our recent work established that the YKD must interact directly with the KD for kinase activation and identified likely KD-YKD contact sites that can be altered to either impair or constitutively activate Gcn2 kinase function in vivo [21].

The extreme C-terminal domain (CTD) of Gcn2 plays multiple roles in kinase regulation, both positive and negative, including dimerization, ribosome binding, and autoinhibition of the KD (Fig. 1A) [1]. Activation of Gcn2 is dependent on KD dimerization [22] in a back-to-back, parallel orientation (Fig. 1B), as described for the active dimer of PKR [23]. While the KD, HisRS region, and CTD are all capable of self-interaction as isolated domains, only the CTD is essential for dimerization and attendant activation of full-length Gcn2 [24,25]. Gcn2 likely dimerizes constitutively through CTD self-interaction [24], and it is possible that the mode of KD dimerization switches from the antiparallel orientation seen in the crystal structure of the inactive conformation of the Gcn2 KD [12] to the parallel, PKR-like mode of dimerization required for kinase function (Fig. 1B) [22] [23]. Recent work elucidated the three-dimensional structure of the CTD dimer, which is evident with some differences in both yeast and mammalian Gcn2 [26].

In addition to dimerization, the CTD mediates ribosome association of Gcn2 (Fig. 1A) [27], which depends on conserved basic residues that mediate RNA binding by the CTD in vitro and are crucial for activation of Gcn2 in vivo [28]. Gcn2 activation also requires *trans*-acting factors Gcn1 and Gcn20, which form a complex that must interact with both the N-terminal “RWD” domain of Gcn2 and translating ribosomes for Gcn2 activation in starved yeast cells [29,30,31,32,33]. These and other findings [34], support the model that Gcn2 is activated by uncharged tRNA that binds first to the decoding center of a translating ribosome and is subsequently transferred to the HisRS domain in Gcn2, with Gcn1/Gcn20 enhancing one or both of these reactions involving uncharged tRNA [33]. However, stable association of mammalian Gcn2 with ribosomes was not observed [26], and it was proposed that the RNA binding activity of the CTD supports tRNA binding by mammalian Gcn2, in the manner described previously for yeast Gcn2 [17].

The yeast Gcn2 CTD also appears to interact with the KD in a manner that impedes kinase activation (Fig. 1A) [17,18], as a mutation that constitutively activates Gcn2 kinase function, *GCN2* ^*c*^-*E803V* (substitutes Glu-803 for Val in the KD) weakens interaction between the isolated KD and CTD and also increases tRNA binding by Gcn2 in vitro [17,18]. Consistent with an autoinhibitory function, eliminating the CTD from mouse Gcn2 activates eIF2α phosphorylation and abrogates stimulation by uncharged tRNA in vitro [35]. The finding that tRNA competed for interaction between the isolated KD and a HisRS-CTD segment of yeast Gcn2 in vitro [17] led to the proposal that the HisRS and CTD domains both dissociate from the KD on tRNA binding. However, complete dissociation of the HisRS domain seems incompatible with the subsequent finding that an N-terminal segment of the HisRS region interacts with the KD and is crucial for Gcn2 activation at a step following tRNA binding, suggesting that this portion of the HisRS domain remains engaged with the KD in the activated state [18]. In addition to the autoinhibitory CTD-KD interaction, the CTD mediates an inhibitory interaction with translation elongation factor eEF1A that can be overcome by uncharged tRNA [36].

While it is clear that tRNA binding to the HisRS domain is required for activation, and a stimulatory interaction of the HisRS-N region with the KD seems likely, it was unclear how tRNA binding might antagonize the autoinhibitory KD-CTD interaction and simultaneously promote stimulatory association of the YKD with the KD. In an effort to answer this question, we identified substitutions in the HisRS region that restore kinase activation by the *gcn2-m2* variant, which harbors substitutions in conserved residues of the predicted HisRS active site cleft that impair tRNA binding in vitro and kinase activation in vivo [15,16,17]. We reasoned that such *m2* suppressors could alter a regulatory surface in the HisRS whose interactions with another domain are modulated by tRNA binding in a manner mimicking the tRNA-bound state of WT Gcn2. Interestingly, the locations of these suppressors led us to identify a regulatory patch predicted to be surface-exposed and proximal to the region in the HisRS domain corresponding to the active site of authentic HisRS, below dubbed the “pseudo-active site”, which can be altered to either activate or impede Gcn2 function. Our finding that certain of the (Gcn^-^) inactivating substitutions strengthen HisRS-CTD interaction without affecting tRNA binding in vitro implies that one stimulatory consequence of tRNA binding is to weaken HisRS-CTD association. This inference leads to an appealing model for how tRNA binding releases the autoinhibitory KD-CTD interaction, promotes YKD-KD association, and thereby activates Gcn2.

## Results

### Identification of Gcd^-^ substitutions in the HisRS domain as suppressors of the *m2* mutation

**Fig 2:**
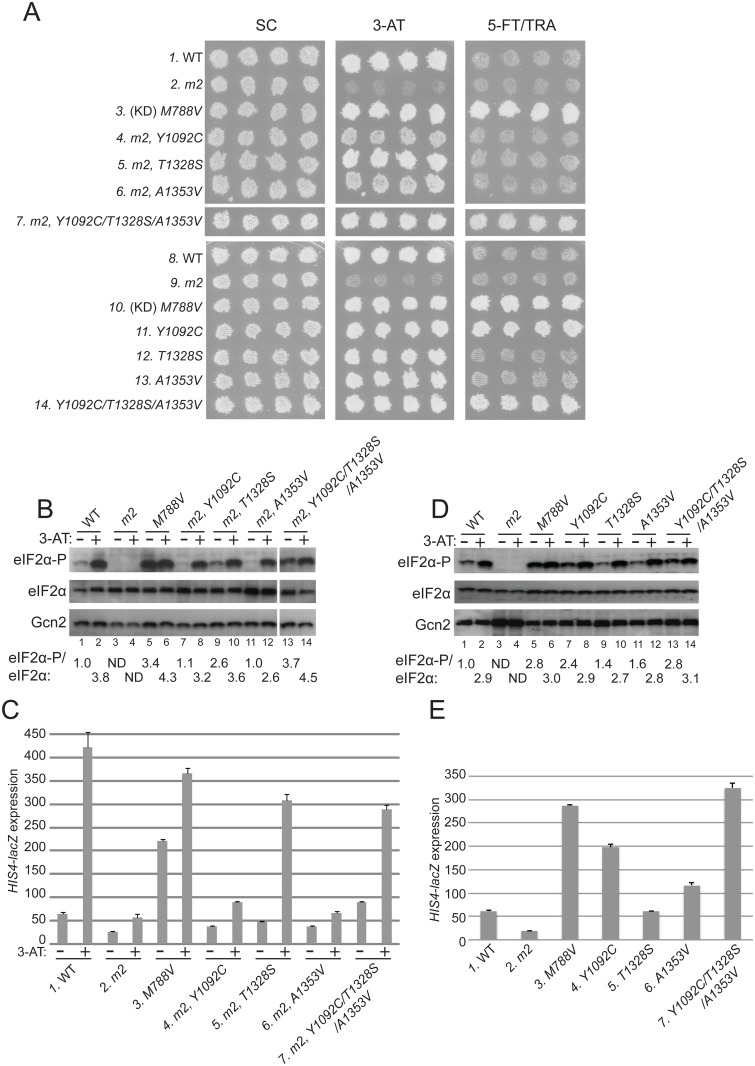
Substitutions in the HisRS domain suppress the *m2* mutation and constitutively activate Gcn2 in vivo. **(A)** Transformants of *gcn2Δ* strain H1149 containing derivatives of low-copy plasmid p722 with wild-type *GCN2*, *gcn2-m2*, *GCN2* ^*c*^-*M788V*, or the indicated mutations affecting residues in the HisRS domain either in combination with *m2* (rows 4–7) or separated from *m2* (rows 11–14) were replica-plated to synthetic complete medium lacking uracil (SC-Ura), SC-Ura plus 30 mM 3-AT, or minimal synthetic medium (SD) supplemented with 0.5 mM 5-FT and 0.125 mM TRA (5FT/TRA) and incubated for 3 d at 30°C. For rows 3 & 10, (KD) signifies a kinase domain substitution by *M788V*. Images were cropped from results obtained from different plates examined in parallel in the same experiment. **(B and D)** Cultures of strains from panel A were grown in liquid SC medium lacking uracil and histidine (SC-Ura-His) to saturation, diluted into fresh SC-Ura-His at A_600_ of ≈0.2, and grown for 6 h at 30°C. 3-AT was added at 10 mM to one culture for 1 h before harvesting (even-numbered lanes). WCEs were resolved by SDS-PAGE and subjected to Western analysis using the indicated specific antibodies and enhanced chemiluminescence to detect immune complexes. (Note that strains examined in panel B harboring suppressor mutations also contain *m2*; whereas *m2* is absent in the strains analyzed in panel D.) Western signals on the upper panel (eIF2α-P) were quantified by scanning densitometry of exposed films using ImageJ software, normalized for the corresponding signals in the middle panel (total eIF2α), and the mean ratios of the two signals (eIF2α-P/eIF2α) calculated from replicate measurements are indicated below the corresponding lanes. Standard errors are less than 6.5% of the mean values shown. The results in lanes 13–14 were cropped from the same Western blot containing lanes 1–12. **(C and E)** Strains from (A) were cultured in both nonstarvation and starvation conditions (C), or only in nonstarvation conditions (E), as described in Materials and Methods, and WCEs were prepared and assayed for β-galactosidase activities. Results are the means and S.E.M.s calculated from three transformants, with activity expressed as nanomoles of *o*-nitrophenyl-β-D-galactopyronoside hydrolyzed per minute per milligram of protein. (Note that strains examined in panel C harboring suppressor mutations also contain *m2*; whereas *m2* is absent in the strains analyzed in panel E.)

In an effort to identify residues in the Gcn2 HisRS domain involved in regulation of kinase function by uncharged tRNA, we randomly mutagenized the coding sequence for the HisRS domain in a plasmid-borne *gcn2-m2* allele and selected for suppressors of the sensitivity to 3-aminotriazole (3-AT) conferred by this allele in yeast cells. The *m2* mutation substitutes two residues in highly conserved motif 2 in the pseudo-active site of the HisRS domain, impairing tRNA binding by Gcn2 in vitro and abolishing activation of Gcn2 kinase function in vivo [15,17]. Defective activation of Gcn2 confers sensitivity to 3-AT, an inhibitor of histidine biosynthesis, by preventing Gcn2-dependent induction of *GCN4* translation and attendant derepression of histidine biosynthetic enzymes under Gcn4 control. Thus, transformants of a *gcn2∆* strain harboring WT *GCN2* or *GCN2* ^*c*^-*M788V*, whose product is constitutively activated [37], grow well on 3-AT medium, whereas *gcn2-m2* transformants do not (Fig. 2A, rows 1–3).

Interestingly, we identified 3 mutations in the HisRS domain that suppress the 3-AT^S^ phenotype of the *m2* mutation, with the strongest growth on 3-AT displayed by the *m2*,*T1328S* mutant (Fig. 2A, 3-AT, rows 4–6 vs. 2). As expected, a mutant allele combining all three suppressors with *m2* also confers a strong 3-AT^R^ phenotype (Fig. 2, row 7). Comparison of the single and triple suppressor alleles at elevated temperature (37°), which exacerbates sensitivity to 3-AT, reveals that combining the suppressor mutations in one allele confers greater resistance to 3-AT than that given by any of the single suppressors (S1 Fig.).

The allele combining all three suppressors with *m2* additionally conferred resistance to a combination of tryptophan analog 5-fluorotryptophan and histidine analog triazolealanine (Fig. 2A, 5-FT/TRA, row 7). The 5FT^R^/TRA^R^ phenotype signifies constitutive, Gcn4-mediated derepression of tryptophan and histidine biosynthetic enzymes, known as the Gcd^-^ phenotype [37]. Accordingly, the *GCN2* ^*c*^-*M788V* allele confers growth on 5-FT/TRA medium, whereas *GCN2^+^* cells, and Gcn^-^ strains like *gcn2-m2*, are sensitive to these analogs (Fig. 2A, rows 1–3). *GCN2* ^*c*^-*M788V* alters the ATP binding pocket of the KD to elevate kinase activity at low levels of uncharged tRNA [12,37]. Thus, it appears that combining all three *m2* suppressors confers constitutive activation of Gcn2, even in the presence of the *m2* mutation.

In accordance with their suppression of the 3-AT^S^ phenotype of *m2*, all three suppressor mutations also restored Gcn2 kinase function under starvation conditions. Western blot analysis of whole cell extracts (WCEs) revealed that 3-AT evokes the expected increase in eIF2α phosphorylated on Ser-51 (eIF2α-P) relative to total eIF2α in *GCN2* cells, whereas *m2* cells have no detectable eIF2α-P; and *M788V* cells display high-level eIF2α with and without 3-AT treatment (Fig. 2B, lanes 1–6). Importantly, each of the suppressor alleles restored 3AT induction of eIF2α-P in *m2* cells, without increasing Gcn2 abundance (Fig. 2B, lanes 7–14). In agreement with its 5FT^R^/TRA^R^ phenotype, the *m2* mutant harboring all three suppressors also displayed a greater than WT level of eIF2α-P in nonstarvation conditions (Fig. 2B, lane 13 vs. 1), indicating constitutive activation of Gcn2. Consistent with these findings, the *m2* suppressors increase expression of a Gcn4-dependent *HIS4-lacZ* reporter [37]. *HIS4-lacZ* expression in 3-AT-starved cells is ~8-fold lower in *m2* versus WT cells, and each of the mutants containing one or more suppressor mutations displays substantially higher reporter expression in 3-AT treated cells compared to that seen in the *m2* single mutant, although only a slight increase was observed for the *A1353V* suppressor (Fig. 2C). The particularly large increases in *HIS4-lacZ* expression observed for the *m2* strains harboring *T1328S* or the triple suppressor mutation are consistent with their marked 3-AT-resistant phenotypes (Fig. 2A).

It is noteworthy that all of the suppressor strains display an induction of eIF2α-P in response to 3-AT treatment (Fig. 2B, lanes 7–14, 3-AT + vs.–), implying that their Gcn2 variants can be activated by uncharged tRNA accumulating in histidine-deprived cells. As demonstrated below, the *m2* mutation reduces, but does not abolish tRNA binding by Gcn2 in vitro. It was possible, therefore, that the suppressor mutations overcome the activation defect of *m2* simply by restoring robust tRNA binding by the HisRS domain. Alternatively, they could increase the ability of low-levels of tRNA bound by the *m2* variant of the HisRS domain to activate Gcn2. If the latter was true, we reasoned that the suppressor mutations should elevate eIF2α phosphorylation when separated from the *m2* mutation in nonstarvation conditions by enabling Gcn2 activation by the low, basal level of uncharged tRNA present in amino acid-replete cells. Consistent with this last prediction, when separated from *m2*, the *Y1092C* and triple suppressor mutations each evoked strong resistance to 5-FT/TRA (Fig. 2A, rows 10,11,14). They also conferred marked increases in eIF2α-P (Fig. 2D, lanes 5,7,13) and *HIS4-lacZ* expression (Fig. 2E, lanes 3,4,7) under nonstarvation conditions, comparable in degree to that given by *GCN2* ^*c*^-*M788V*. The *A1353V* single mutation conferred smaller increases in eIF2α-P accumulation and derepression of *HIS4-lacZ* (Fig. 2D, lane 1 vs. 11; Fig. 2E, column 1 vs. 6). Thus, it appears that the triple suppressor mutation, *Y1092C*, and to a lesser extent *A1353V* increase the ability of low-level uncharged tRNA present in nonstarved cells to stimulate Gcn2 kinase function. The triple mutant was chosen as the exemplar Gcd^-^ variant for subsequent biochemical studies described below.

Surprisingly, despite being the most effective suppressor of *m2*, the *T1328S* single mutation produced only a slight increase in eIF2α-P (Fig. 2D) and no increase in resistance to 5-FT/TRA or *HIS4-lacZ* expression (Fig. 2A & E). Thus, although *T1328S* restores robust activation of the *m2* variant in starved cells, it does not appreciably activate otherwise WT Gcn2 in nonstarvation conditions.

### *m2* suppressors and *GCN2* ^*c*^ substitutions alter residues in the HisRS domain pseudo-active site

**Fig 3:**
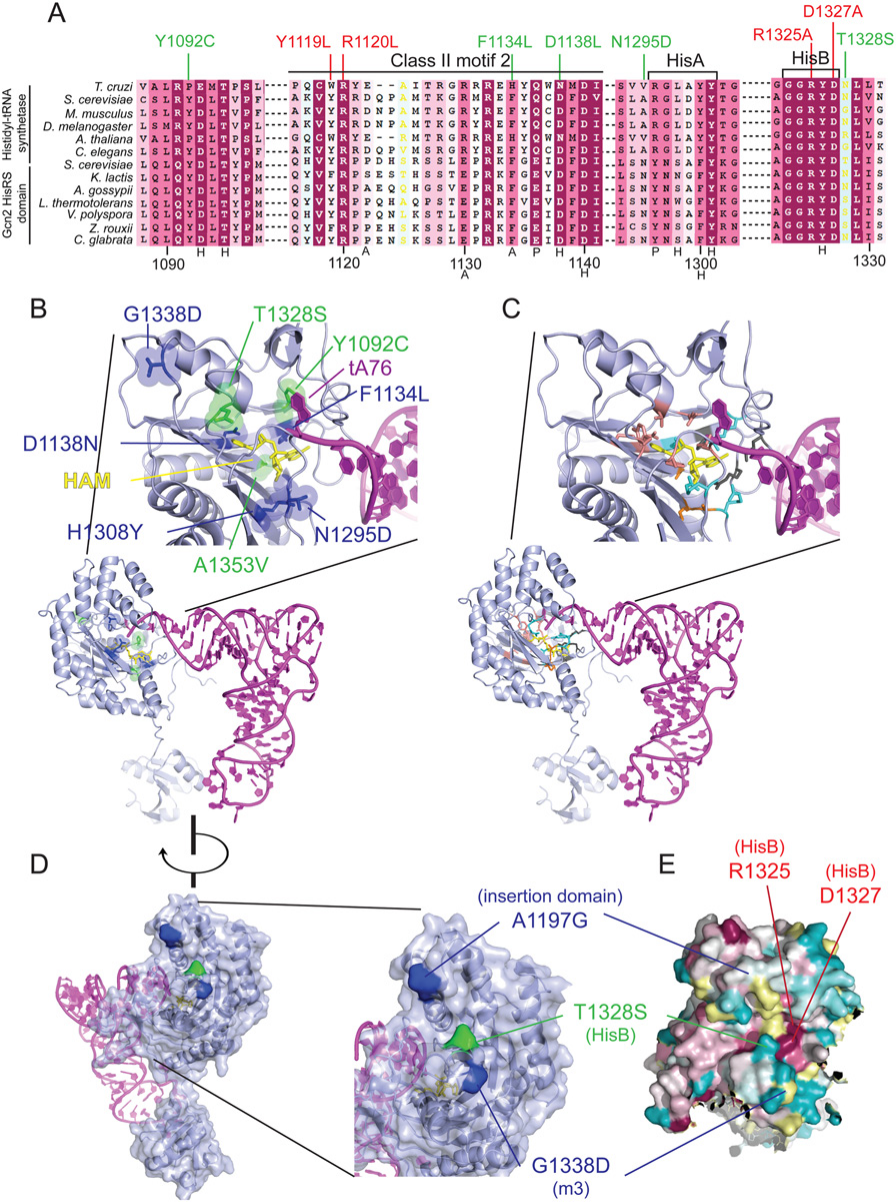
Gcd^-^ substitutions in the HisRS domain cluster near the predicted pseudo-active site. **(A)** Excerpt of the multiple sequence alignment shown in S2 Fig. of authentic HisRSs and HisRS domains from fungal Gcn2 sequences, identified on the left by abbreviations of their species of origin, built using the MUSCLE program. Residues are colored according to evolutionary sequence variation as analyzed with the CONSURF on-line server, with dark magenta corresponding to the most conserved residues. Yellow color indicates residues for which the data were insufficient to calculate a reliable conservation grade. Numbering corresponds to residue positions in full-length *S*. *cerevisiae* Gcn2 (residues 1088–1331). Regions of predicted conserved motifs are denoted above the *T*. *cruzi* HisRS sequence. Substitutions conferring Gcn^-^ or Gcd^-^ phenotypes are shown in red and green, respectively. Residues interacting directly with HAM in *T*. *cruzi* HisRS are indicated by black letters below the sequence, with H/P/A signifying interaction with histidyl/phosphate/adenine moieties, respectively. **(B)** Localization of Gcd^−^ substitutions in the predicted 3-D structure of a monomer of the Gcn2 HisRS domain in complex with an uncharged tRNA, based on the crystal structure of *T*. *cruzi* authentic HisRS (PDB: 3HRK) and co-crystal structure of *S*. *cerevisiae* AspRS-tRNA^Asp^ (Materials and Methods). Gcd^-^ substitutions identified here are in green, while previously identified *GCN2* ^*c*^ substitutions are in blue. **(C)** The locations of *T*. *cruzi* HisRS (PDB: 3HRK) residues interacting directly with the histidyl/phosphate/sugar/adenine moieties of HAM (in yellow) are indicated in salmon/cyan/orange/dark gray, respectively. **(D)** Localization of surface-exposed Gcd^-^ substitutions on the predicted 3-D structure of the HisRS domain, colored as in (B). **(E)** The degree of evolutionary conservation of Gcn2 HisRS domain residues shown in S2 Fig. was projected onto the 3-D structure of *T*. *cruzi* authentic HisRS monomer using the CONSURF program, with magenta/dark cyan corresponding to the most/least conserved residues, respectively, and yellow residues corresponding to indeterminate conservation.

To evaluate the locations of the *m2* suppressors in the predicted structure of the HisRS domain, we constructed a multiple sequence alignment of Gcn2 HisRS domain sequences from various fungal species (S2 Fig.), and also an alignment of a subset of these HisRS domain sequences with authentic HisRS enzymes from diverse eukaryotic species (S3 Fig.). The latter reveals regions of considerable sequence similarity between authentic HisRS and the Gcn2 HisRS domains spanning the region extending from motifs 1 and 2, conserved in all class II aminoacyl tRNA synthetases, portions of the insertion domain between motifs 2 and 3 and the HisA and HisB motifs unique to HisRS enzymes, and the N-terminal half of class II motif 3. Interestingly, the three *m2* suppressors *Y1092C*, *T1328S*, and *A1353V* alter residues in the vicinity of motif 2, HisB, and within motif 3, respectively (Fig. 3A and S3 Fig.). Conserved residues of these motifs include active site residues that directly contact different moieties of the intermediate HAM formed in the first step of tRNA aminoacylation (Fig. 3A and S3 Fig., residues labeled with H (histidyl), P (phosphate), S (sugar), or A (adenine)). These critical residues are color-coded in the “ribbons” depiction of the crystal structure of the *T*. *cruzi* HisRS-HAM complex shown in Fig. 3C (salmon (H), cyan (P), orange (S), or dark gray (A)). Interestingly, six *GCN2* ^*c*^ mutations described previously [37] also alter residues located in or nearby the conserved HisRS motifs in the primary sequence, including *F1134L* and *D1138N* (motif 2), *A1197G* (insertion domain), *N1295D* and *H1308Y* (near HisA), and *G1338D* (motif 3) (Fig. 3A and S3 Fig.).

Because the structure of the Gcn2 HisRS domain is unknown, we used the sequence alignment between Gcn2 and authentic HisRSs (S3 Fig.) and the crystal structure of *T*. *cruzi* authentic HisRS to predict the locations of *m2* suppressors and *GCN2* ^*c*^ substitutions in the three-dimensional structure of the Gcn2 HisRS domain (Fig. 3B). It is striking that all three *m2* suppressors and 5 of the 6 previously identified *GCN2* ^*c*^ mutations alter residues within, or in proximity to, the pseudo-active site of the HisRS domain (green residues: *m2* suppressors; blue residues previously known *GCN2* ^*c*^ mutations). In fact, several mutations alter residues corresponding to amino acids in HisRS that make direct contacts with the adenine (*F1134L*), histidyl (*D1138N*), or ribose (*A1353V*) moiety, while others are located only one or two residues away in the polypeptide chain from amino acids contacting the histidyl (*Y1092C* and *T1328S*) or phosphate (*N1295*) moiety of HAM (Fig. 3A, S3 Fig.; cf. Fig. 3B-C). In the cases of *T1328S*, *A1197V* and *G1338D*, these residues are predicted to be surface-exposed and (at least for T1328S and G1338D) in proximity to one another (Fig. 3D-E) at the “top” of the predicted pseudo-active site cleft of the Gcn2 HisRS domain (Fig. 3B).

The predicted locations of these last three substitutions led us to consider a model in which this surface of the HisRS domain interacts with another region in Gcn2 to regulate kinase function in a manner that is modulated by binding of uncharged tRNA to the pseudo-active site. In this view, the putative regulatory interaction involving this patch of the HisRS domain would be altered by the Gcd^-^ *m2* suppressors and *GCN2* ^*c*^ substitutions mapping in the HisRS domain in a way that mimics the effect of uncharged tRNA binding to the pseudo-active site of the WT Gcn2 HisRS domain.

### Identification of Gcn^-^ substitutions in the predicted HisRS pseudo-active site

We reasoned that if the foregoing hypothesis is correct, then it should be possible to isolate Gcn^-^ substitutions affecting the same exposed surface of the HisRS domain altered by the *m2* suppressor T1328S and Gcd^-^ substitution G1338D, but with the opposite effect on its putative regulatory interactions with other Gcn2 domains. To test this idea, we first determined the degree of sequence conservation of residues on this face of the HisRS domain by projecting the sequence conservation scores obtained from the alignment of fungal Gcn2 HisRS domains (S2 Fig.) onto a surface representation of the crystal structure of *T*. *cruzi* HisRS (Fig. 3E). We then determined the phenotypes conferred by substituting two highly conserved residues, Arg-1325 and Asp-1327, which are surface exposed and located in proximity both to one another and the residues altered by T1328S and G1338D (Fig. 3D-E).

**Fig 4:**
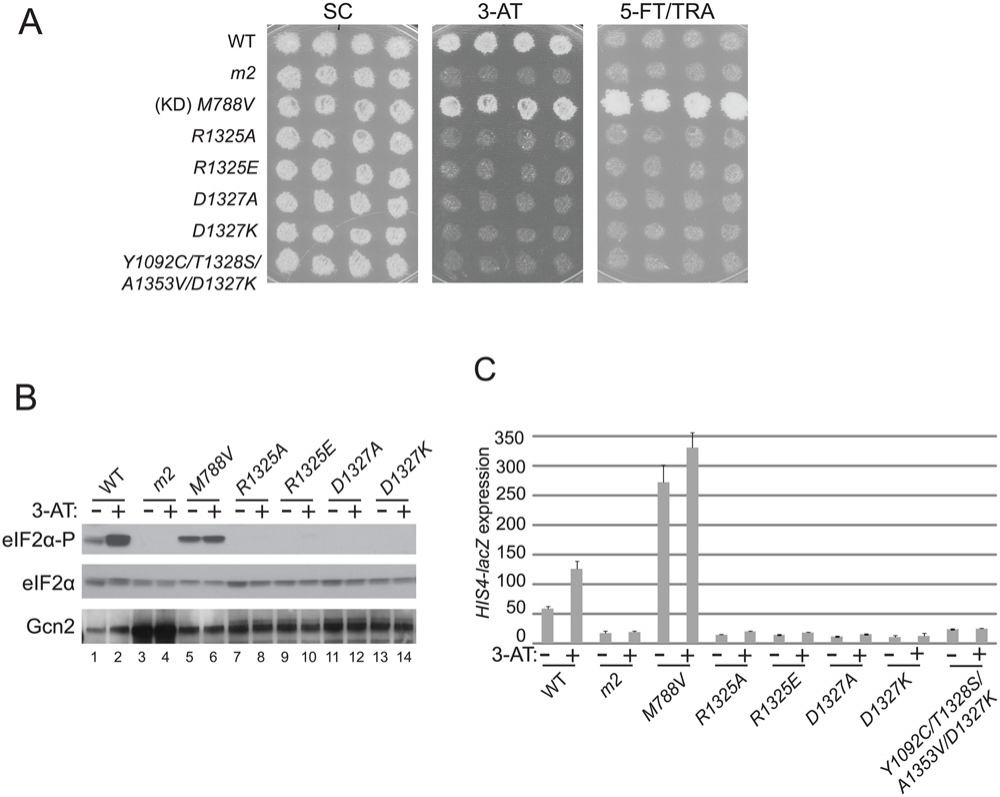
Mutations of conserved surface-exposed residues of the Gcn2 HisRS domain that impair activation of Gcn2 in vivo. **(A)** Growth phenotypes of transformants of *gcn2Δ* strain H1149 containing the indicated plasmid-borne *GCN2* alleles were analyzed as in Fig. 2A. **(B)** Cultures of strains from panel A were analyzed for levels of eIF2α-P as in Fig. 2B. **(C)** *HIS4-lacZ* expression was analyzed in strains from (A) as in Fig. 2C.

Strikingly, substitutions of Arg-1325 with Ala or Glu (*R1325A*, *R1325E*) and substitutions of Asp-1327 with Ala or Lys (*D1327A*, *D1327K*) completely abrogate Gcn2 function. Thus, all four substitutions confer strong sensitivity to 3-AT (Fig. 4A), eliminate detectable eIF2α-P in both nonstarvation and starvation conditions (Fig. 4B), and evoke low basal expression of the *HIS4-lacZ* reporter at levels comparable to, or even below, that given by the *m2* mutation (Fig. 4C); and all of these Gcn^-^ phenotypes occur without any reduction in the level of Gcn2 itself (Fig. 4B). These findings are consistent with the possibility that highly conserved residues Arg-1325 and Asp-1327 are critical constituents of a regulatory patch exposed on the surface of the HisRS domain near the pseudo-active site cleft (Fig. 3E).

Interestingly, a mutant combining the strong Gcn^-^ mutation *D1327K* with the Gcd^-^ triple *m2* suppressor *Y1092C/T1328S/A1353V* described above exhibits a 3-AT-sensitive phenotype (Fig. 4A) and a defect in derepression of *HIS4-lacZ* expression (Fig. 4C) nearly indistinguishable from those seen for the *D1327K* single mutant, indicating that the strong activation defect conferred by *D1327K* is epistatic to the constitutively activating phenotype of the Gcd^-^ suppressor substitutions.

### Gcd^-^ *m2* suppressors and Gcn^-^ substitutions of Asp-1327 in the HisRS domain do not alter tRNA binding by Gcn2

**Fig 5:**
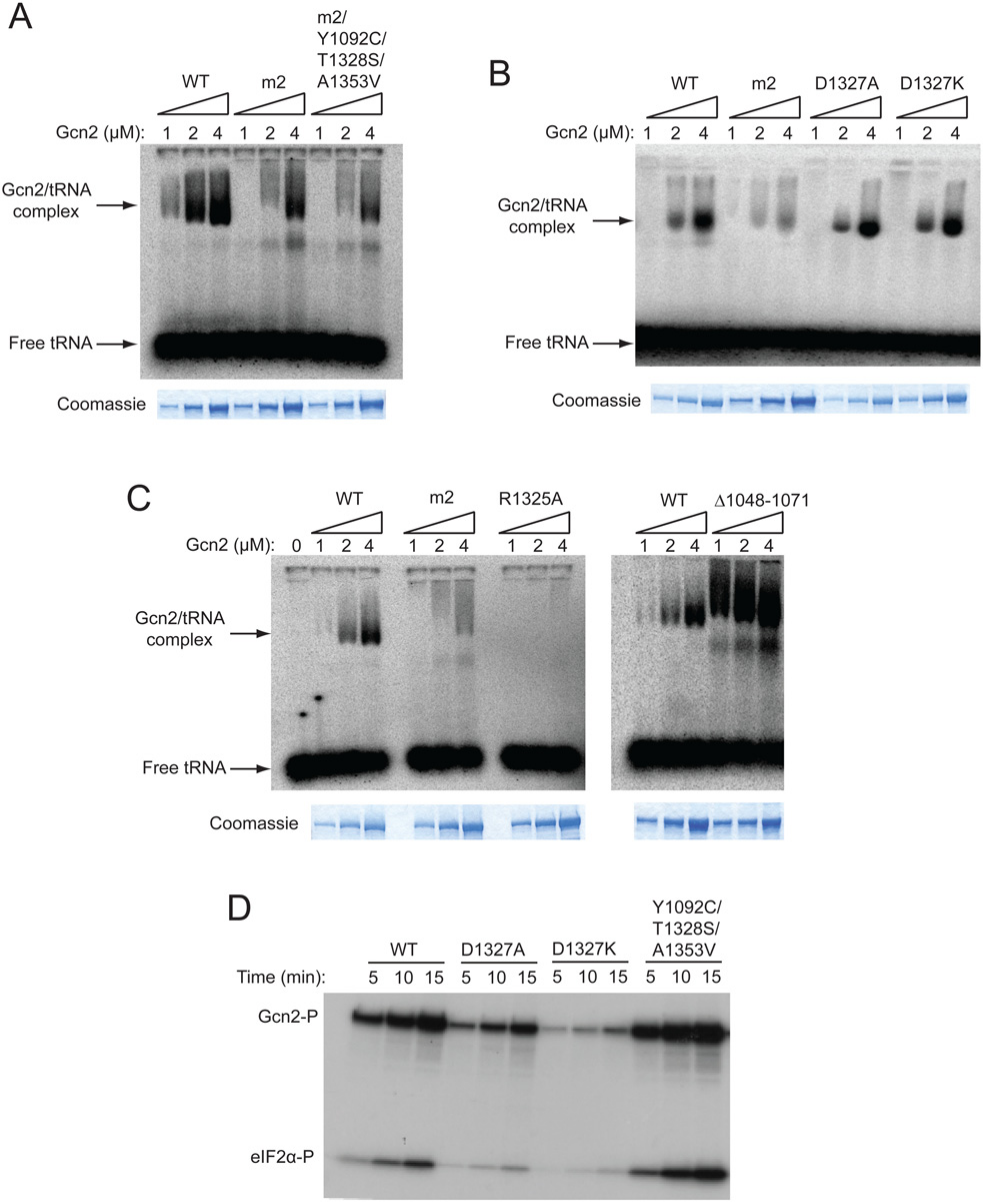
A subset of Gcn^-^ substitutions of conserved surface-exposed residues in the HisRS domain impair kinase activity but not tRNA binding by purified Gcn2 in vitro. **(A, B and C)** The indicated purified Gcn2 proteins were incubated with [^32^P]-labeled total yeast tRNA in 20 μL of GMSA buffer. The Gcn2-tRNA complexes were resolved by electrophoresis through a 1% agarose gel in 1×MOPS buffer (1.5h, 100 V), transferred to a nitrocellulose membrane and visualized by autoradiography. Unbound [^32^P]-tRNA, which has a higher mobility, was present at essentially identical amounts in each lane at levels ~15-fold higher than the WT Gcn2/tRNA complexes formed at 4μM. The purified Gcn2 proteins used in the assays were visualized by staining with Coomassie brilliant blue following separation by SDS-PAGE (images labelled Coomassie). **(D)** The indicated Gcn2 proteins (ca. 0.25 μg) were incubated with 3 μCi of [γ-^32^P]ATP (6000 Ci/mmol, Amersham), 1 μg of recombinant eIF2α−ΔC purified from *E*. *coli*, and 0.5 μg of bovine serum albumin in 20 μL of kinase assay buffer at 30^°^C for the indicated times. Samples were resolved by 8%–16% SDS—PAGE and subjected to autoradiography. Positions of autophosphorylated Gcn2 (Gcn2-P) and phosphorylated eIF2α−ΔC (eIF2α-P) are indicated.

We proposed above that the Gcd^-^ substitutions identified as *m2* suppressors restore Gcn2 kinase function to the *m2* variant by altering a regulatory interaction of the HisRS domain in a way that mimics the effect of uncharged tRNA and allows for Gcn2 activation at low levels of bound tRNA. To bolster this view and eliminate the alternative possibility that they simply overcome the effect of *m2* of impairing tRNA binding, we purified the *gcn2-m2* product and the Gcn2 variant harboring the *m2* substitutions in combination with all three suppressor substitutions, and compared them to WT Gcn2 for binding [^32^P]-labeled total tRNA using a gel mobility shift assay to detect Gcn2-tRNA complexes. In accordance with previous results [15,17], the *m2* product displayed an obvious defect in tRNA binding compared to WT Gcn2; however, unlike the results of our previous studies, it retained appreciable tRNA binding activity (Fig. 5A). (This disparity in results might be attributable to the fact that, unlike the gcn2-m2 protein examined here, this variant was unstable and subject to degradation when purified from a different yeast strain used in previous studies [17].) The fact that *m2* does not abolish tRNA binding in vitro but completely impairs activation of Gcn2 in vivo might indicate that the *m2* substitutions in the pseudo-active site cleft impair a regulatory interaction of the HisRS domain with another region in Gcn2 in addition to reducing tRNA binding. Importantly, the presence of all three suppressors in a quadruple mutant harboring the *m2* substitutions did not increase the tRNA binding activity compared to that measured for gcn2-m2 (Fig. 5A). These findings are consistent with our conclusion that the suppressor substitutions restore eIF2α-P formation by enhancing kinase function at the low tRNA occupancy permitted by the *m2* substitutions, rather than restoring high-level tRNA binding to the HisRS domain.

We also examined whether Gcn^-^ substitutions affecting the conserved, surface-exposed residues Arg-1325 and Asp-1327 proximal to the pseudo-active site affect tRNA binding. Importantly, we saw little or no effect on tRNA binding by the Gcn^-^ substitutions D1327A and D1327K (Fig. 5B), implying that they impair activation of Gcn2 by disrupting the ability of bound tRNA to trigger activation of kinase function rather than reducing the amount of bound tRNA. By contrast, the Gcn^-^ substitution of Arg-1325, R1325A, abolished tRNA binding by Gcn2 (Fig. 5C), making it likely that substitutions of this residue impair Gcn2 activation by reducing the level of bound tRNA, although they could also disrupt the proposed regulatory interactions involving the HisRS pseudo-active site. In accordance with previous findings, deletion of HisRS residues 1048–1071 evokes a greater than WT level of tRNA binding by Gcn2 (Fig. 5C), supporting our previous conclusion that removing this N-terminal segment of the HisRS domain impairs Gcn2 activation by disrupting a stimulatory interaction of the tRNA-bound HisRS domain with the KD rather than impairing tRNA binding by Gcn2 [18]. Based on its reduced electrophoretic mobility, the gcn2-∆1048–1071 variant might also exhibit a less compact conformation compared to WT Gcn2.

We wished to confirm that the key regulatory mutations of interest, the Gcn^-^ substitutions D1327A and D1327K, and the Gcd^-^ triple substitution Y1092C/T1328S/A1353V, alter Gcn2 kinase activity in vitro in the manner predicted by their phenotypes in vivo. To this end, we conducted in vitro kinase assays with the relevant purified Gcn2 proteins using [γ-^32^P]-ATP and a truncated form of recombinant eIF2α as substrates, and employed SDS-PAGE/autoradiography to detect the reaction products. It was shown previously that WT Gcn2 displays similar kinase activity whether purified from starved or nonstarved cells, but that the *m2* mutation reduces kinase activity in vitro, indicating that WT Gcn2 becomes activated in vitro by deacylated tRNA in cell lysates prior to purification [16]. Thus, although yeast Gcn2 cannot be activated further by adding tRNA to kinase assays, the activity levels of Gcn2 variants with HisRS domain substitutions should reflect their abilities to be activated by tRNA during purification. Consistent with their Gcn^-^ phenotypes, the D1327A and D1327K variants also exhibit substantially reduced autophosphorylation and eIF2α substrate phosphorylation activities in vitro (Fig. 5D). Moreover, the Gcd^-^ variant Y1092C/T1328S/A1353V exhibits an obvious increase in kinase activity relative to WT Gcn2 (Fig. 5D).

### Gcn^-^ substitutions of Asp-1327 strengthen HisRS interaction with the CTD

Our finding that Gcn^-^ variants D1327A/D1327K are completely defective for Gcn2 activation (Fig. 4A) but retain robust tRNA binding activity (Fig. 5B) made them good candidates for mutations that alter a regulatory interaction of the HisRS region that mediates allosteric activation of kinase function by uncharged tRNA. Previously, we demonstrated that distinct segments of the isolated HisRS domain interact with the isolated KD or CTD of Gcn2 [18]. As noted above, the N-terminal HisRS segment (HisRS-N) interacts with the KD and the *Δ1048–1071* deletion in this region impairs Gcn2 activation without reducing tRNA binding, thus identifying a stimulatory HisRS-N/KD interaction [18]. Moreover, the C-terminal HisRS segment (HisRS-C) was shown to interact with the CTD [18], and as it encompasses Asp-1327, we hypothesized that the Gcn^-^ D1327A/D1327K substitutions impair Gcn2 function by altering the HisRS-CTD interaction.

**Fig 6:**
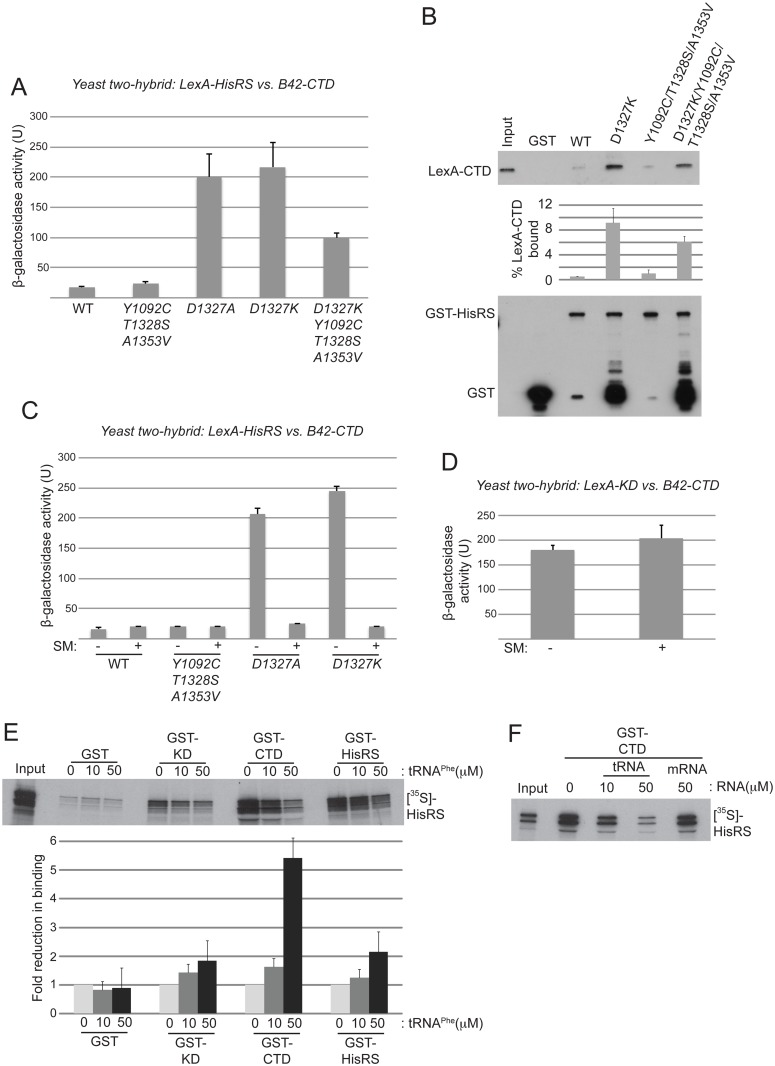
Gcn^-^ substitutions D1327A/D1327K strengthen HisRS/CTD domain interaction in a manner antagonized by uncharged tRNA. **(A)** Yeast two-hybrid analysis of domain interactions. Strain EGY48 carrying the *lexAop-lacZ* reporter plasmid was co-transformed with plasmids encoding lexA-HisRS (Gcn2 residues 970–1497) (with or without the indicated substitutions) and B42-CTD (Gcn2 residues 1497–1659). Strains were cultured and β-galactosidase activities were measured in WCEs. **(B)** *In vitro* binding of LexA-CTD (Gcn2 residues 1498–1659), expressed in yeast, to GST fusion proteins containing the Gcn2 HisRS domain (residues 970–1497), either WT or containing the indicating mutations. Aliquots of nuclease-treated whole-cell extracts (500 μg of total protein) from transformants of yeast *gcn2*Δ strain HQY132 carrying plasmids containing the LexA-CTD fusion protein were incubated with approximately equivalent amounts of GST or GST-HisRS (970–1497) fusion proteins immobilized on glutathione-Sepharose beads. After extensive washing, LexA fusion proteins bound to the GST proteins were resolved by SDS-PAGE and detected by immunoblot analysis with anti-LexA (Top) and anti-GST (bottom) antibodies. Results from quantification of LexA blots from replicate experiments are summarized in the histogram below as mean percentages of input amounts recovered in the pull-downs. **(C)** Yeast two-hybrid analysis as shown in (A). Samples for β-galactosidase assay were collected from cells under starvation (+) or nonstarvation (-) conditions as described in Materials and Methods. The results shown are averages of activities from three or more individual transformants. **(D)** Yeast two-hybrid analysis of CTD and KD interaction domains in Gcn2. Plasmids pHQ311 and pHQ428 encoding respectively the lexA-CTD(1498–1659) and B42-KD(720–999) segments were co-transformed into strain EGY48 carrying the *lexAop-lacZ* reporter plasmid. Samples for β-galactosidase assay were collected from cells under starvation (+) or nonstarvation (-) conditions. The results shown are averages of activities from three or more individual transformants. **(E and F)** *In vitro* binding of Gcn2 [^35^S]-HisRS(970–1497) segment translated *in vitro* to GST fusion proteins containing the Gcn2 KD (568–998), CTD (1498–1659) or HisRS (970–1497) domains. RNA (tRNA^Phe^ or mRNA) was added to the reaction to the indicated final concentration. The mean fold-reductions in pull-downs of [^35^S]-HisRS(970–1497) on tRNA^Phe^ addition were calculated from replicate experiments and presented in the histogram below the gel.

We obtained evidence supporting this hypothesis using the yeast two-hybrid assay. In agreement with previous results [24], a LexA-fusion to the WT Gcn2 HisRS domain shows little interaction with a fusion of the B42 activation domain to the CTD. Remarkably, introducing Gcn^-^ substitutions D1327A or D1327K into the lexA-HisRS fusion greatly enhanced this two-hybrid interaction (Fig. 6A). By contrast, the Gcd^-^ triple substitution Y1092C/T1328S/A1353V had no significant effect on the HisRS/CTD interaction when introduced into otherwise WT lexA-HisRS. Interestingly, however, these Gcd^-^ substitutions diminished the enhanced two-hybrid interaction conferred by the D1327K Gcn^-^ substitution (Fig. 6A).

In an effort to confirm the two-hybrid findings, we examined in vitro interaction of a LexA-CTD fusion expressed in yeast cells with immobilized GST fusions containing mutant or WT HisRS segments purified in yeast. As both the HisRS and CTD segments have RNA binding activity [17,28], the reactants were treated with micrococcal nuclease to eliminate indirect association between these segments bridged by RNA. Paralleling the two-hybrid results, the D1327K substitution greatly increased binding of LexA-CTD to GST-HisRS compared to the low-level binding observed for both WT GST-HisRS and the variant harboring the Gcd^-^ triple substitution Y1092C/T1328S/A1353V; and introducing the Gcd^-^ triple substitution reduced binding by the D1327K variant (Fig. 6B). It could be argued that the truncated species of the GST-HisRS-D1327K fusion that are not observed for WT GST-HisRS (lower blot, lane 3 vs. 2) mediate the relatively greater binding of LexA-CTD by GST-HisRS-D1327K; however, this interpretation is inconsistent with the fact that the GST-HisRS fusion harboring substitutions D1327K/Y1092C/T1328S/A1353V contains even greater levels of the truncated species but binds relatively smaller amounts of LexA-CTD compared to GST-HisRS-D1327K (Fig. 6B, lane 5 vs. 3). Our finding that Y1092C/T1328S/A1353V does not reduce the HisRS/CTD interaction when introduced into the otherwise WT HisRS segment might be explained by proposing that a physiologically relevant interaction between the WT HisRS and CTD domains cannot be captured outside of the context of full-length Gcn2 in two-hybrid or pull-down assays unless the HisRS segment contains the Gcn^-^ substitutions D1327A/D1327K that stabilize HisRS/CTD association. Together, the findings in Fig. 6A-B suggest that the D1327A/D1327K substitutions impair activation of Gcn2 by strengthening the HisRS-CTD domain interaction, while the Gcd^-^ substitution Y1092C/T1328S/A1353V activates Gcn2 by weakening HisRS/CTD association.

### Evidence that tRNA antagonizes HisRS/CTD interaction

A corollary of this last conclusion is that the HisRS-CTD domain interaction in WT Gcn2 stabilizes the inactive conformation of Gcn2, which would persist constitutively in the Gcn^-^ mutants D1327A/D1327K with attendant impairment of Gcn2 activation. If so, then binding of uncharged tRNA to the HisRS region might be expected to weaken the HisRS-CTD domain interaction as one means of activating Gcn2. Supporting this possibility, we found that the enhanced HisRS-CTD two-hybrid interactions conferred by D1327A or D1327K in vivo were abolished by starving the cells for isoleucine/valine by treatment with sulfometuron methyl (SM) (Fig. 6C), an inhibitor of the *ILV2*-encoded biosynthetic enzyme, which is known to increase the level of uncharged tRNA^Ile^ and tRNA^Val^ and activate Gcn2 in vivo [15,38]. By contrast, the previously demonstrated two-hybrid interaction between LexA-KD and B42-CTD fusion proteins was unaffected by SM treatment (Fig. 6D), as was expression of the two-hybrid reporter conferred by the LexA-B42 activator. These findings are consistent with the idea that accumulation of uncharged tRNA^Ile^ and tRNA^Val^ and their attendant increased binding to the LexA-HisRS-D1327A and LexA-HisRS-D1327K fusions weakens the ability of these LexA-HisRS proteins to form complexes with the B42-CTD fusion in vivo. They also support the idea that the gcn2-D1327A and gcn2-D1327K variants are defective for a regulatory interaction with the CTD that is normally disrupted by uncharged tRNA binding to the HisRS domain.

To provide additional evidence that tRNA binding to the HisRS domain reduces its ability to interact with the CTD, we examined the effect of tRNA on this interaction in vitro. Consistent with previous results [18,24], [^35^S]-methionine labeled HisRS fragment can be pulled down with immobilized GST fusions containing the Gcn2 KD, CTD or HisRS region itself, with the last interaction reflecting dimerization of the HisRS domain [18] (Fig. 6E). Addition of increasing amounts of purified yeast tRNA^Phe^ reduced interaction of the [^35^S]-HisRS fragment with all three GST fusions; however, the magnitude of the reduction was larger for GST-CTD (~5.4-fold) compared to GST-KD (~1.8-fold) or GST-HisRS (~2.1-fold). Moreover, interaction of [^35^S]-HisRS with GST-CTD was unaffected by an equivalent concentration of an unstructured model mRNA [39] (Fig. 6F), suggesting specificity for tRNA in weakening the HisRS-CTD domain interaction. Together, these findings provide evidence that a tight interaction between the HisRS and CTD domains favors the inactive conformation of Gcn2 and that tRNA binding to the HisRS domain activates Gcn2 at least partly by weakening the HisRS-CTD interaction. As discussed below, based on previous findings indicating that direct interaction of the CTD with the KD contributes to the latency of Gcn2 kinase function [18], we propose that the HisRS-CTD interaction helps to stabilize this inhibitory CTD-KD interaction in a manner that is diminished by uncharged tRNA binding to the HisRS domain in amino acid-starved cells.

### Gcd^-^ triple substitution increases protease sensitivity of full-length Gcn2

The model alluded to above envisions that the non-activated state of Gcn2 is characterized by domain interactions between the HisRS-C and CTD, and between the CTD and KD, which are destabilized by tRNA binding to the HisRS domain to evoke the activated state. We reasoned that the Y1092C/T1328S/A1353V Gcd^-^ substitutions, which destabilize the HisRS/CTD interaction and confer constitutive activation of Gcn2, would evoke a conformational change in full-length Gcn2 that mimics the activated, tRNA-bound state of WT Gcn2. Supporting this possibility, we found that the Gcd^-^ triple substitution increases the sensitivity of full-length purified Gcn2 to digestion by elastase, reducing the amount of full-length protein remaining after a fixed time of incubation compared to WT Gcn2 or the Gcn^-^ variant D1327K (S4 Fig.). Trypsin digestion also reduced the amounts of the largest intermediates in addition to the full-length protein for the Y1092C/T1328S/A1353V variant compared to the WT and D1327 proteins (S4 Fig.). Judging by the amount of full-length Gcn2 remaining after partial digestion, the Gcn^-^ variant D1327K appears to be somewhat less sensitive than WT Gcn2 to protease digestion, consistent with the tighter HisRS/CTD domain interaction conferred by D1327K (Fig. 6A-C); although this difference is less pronounced than that between WT and the Y1092C/T1328S/A1353V variant (S4 Fig.). These results support the idea that activation of Gcn2 by the Y1092C/T1328S/A1353V substitutions involves the elimination of inhibitory domain interactions, which favors a less compact conformation of Gcn2. It should be noted that in separate experiments we observed a decrease in protease sensitivity of WT Gcn2 on addition of excess tRNA^Phe^. While this result is ostensibly at odds with the notion that tRNA binding evokes a more extended, protease-sensitive conformation of Gcn2, it seems possible that contacts between tRNA and the HisRS or CTD domains would reduce protease access to these Gcn2 segments and compensate for loss of protein-domain interactions in the tRNA-free state of Gcn2.

## Discussion

In this study, we used a genetic approach to identify a novel regulatory surface in the HisRS domain of Gcn2, juxtaposed to the pseudo-active site cleft where tRNA binds, which participates in the activation of kinase function in amino acid starved cells through its association with the Gcn2 CTD. One of the residues belonging to this regulatory surface, Thr-1328, was identified by isolating suppressors of the *m2* lesion in motif 2 of the HisRS domain, a mutation that reduces tRNA binding to Gcn2 and abolishes activation of kinase function in starved cells. Two other *m2* suppressors alter residues Tyr-1092 and Ala-1353 located within the pseudo-active site cleft. The suppressor substitution Y1092C, as well as the combination of all three suppressor substitutions in the same protein, confer constitutive activation of Gcn2 function in the absence of the *m2* substitutions—the Gcd^-^ phenotype—and we showed that the triple substitution does not suppress the tRNA binding defect evoked by *m2*. Hence, rather than influencing the level of tRNA binding, we propose that these mutations evoke a conformational change in the HisRS domain that mimics the consequences of tRNA binding to the WT HisRS region, which then mediates activation of the adjacent KD. In this view, the *m2* suppressors allow rearrangement of Gcn2 to the active conformation at a lower occupancy of tRNA in the HisRS pseudo-active site. This alteration would compensate for the reduced affinity for tRNA of the *m2* variant, and in the cases of Y1092C and A1353V allow for activation of otherwise WT Gcn2 by the basal level of uncharged tRNA in non-starved cells to produce the Gcd^-^ phenotype. Our conclusion above that *T1328S* corrects the activation defect conferred by *m2* but does not appreciably activate otherwise WT Gcn2, ie. *T1328S* is not Gcd^-^, indicates that replacing Thr with Ser at this position promotes tRNA binding only in the context of the *m2* alterations of the HisRS pseudo-active site. This restricted efficacy of *T1328S* is consistent with the fact that Thr-1328 is not evolutionarily conserved in Gcn2 HisRS domains, and is even substituted with Ser in some species (S2 Fig.). Given that the *m2* lesion abolishes Gcn2 activation in vivo but only reduces tRNA binding in vitro, the *m2* substitutions might also impair regulatory interactions of the HisRS domain that can be compensated by the *m2* suppressors.

A second line of evidence supporting this model is that all 6 previously identified *GCN2* ^*c*^ mutations affecting the HisRS domain [37] involve substitutions mapping within, or proximal to, the pseudo-active site cleft. These mutations were identified by screening randomly mutagenized *GCN2* alleles for the Gcd^-^ phenotype, rather than selecting for *m2* suppressors. The striking clustering of these 6 *GCN2* ^*c*^ substitutions in the predicted structure of the HisRS domain suggests that the pseudo-active site cleft is the key regulatory hub in this domain. The *GCN2* ^*c*^ mutations *D1138N* and *F1134L* alter residues in proximity to those substituted by the *m2* suppressors *Y1092C* and *A1353V* within the pseudo-active site (Fig. 3B) and thus, according to our model, would evoke a rearrangement of the active site that mimics the effect of tRNA binding. The *GCN2* ^*c*^ mutations *G1338D* and *A1197G* introduce substitutions proximal to the active site, but located on a distinct surface, with Gly-1338 nearly adjacent on that surface to Thr-1328 (altered by the *m2* suppressor *T1328S*). We envision that this surface patch in the WT HisRS domain communicates with the pseudo-active site and is remodeled by tRNA binding in a manner mimicked by the Gcd^-^ substitutions G1338D, A1197G, and *m2* suppressor T1328S.

A third line of genetic evidence supporting this model came from making targeted alanine substitutions of two HisRS domain residues that are invariant among Gcn2 homologs and exposed on the putative regulatory surface that circumscribes the Gcd^-^ substitutions G1338D, A1197G, and *m2* suppressor T1328S. Ala or Lys substitutions of the highly conserved residue D1327 completely abolish Gcn2 function in vivo while retaining robust tRNA-binding activity in vitro. It is remarkable that Gcn^-^ and Gcd^-^ substitutions of nearby residues belonging to this patch of the HisRS surface have opposite effects on Gcn2 activation. We envision that the Gcn^-^ substitutions D1327K/D1327A either impede the proposed conformational remodeling of this surface patch induced by tRNA binding or alter the affinity of the remodeled surface for its binding partner within Gcn2.

The latter possibility is supported by our finding that Gcn^-^ substitutions D1327K/D1327A enhance interaction between the HisRS and CTD domains, whereas the Gcd^-^ triple substitution Y1092C/T1328S/A1353V partially reverses this effect in the quadruple mutant also containing D1327K. These findings imply that tight binding between the HisRS regulatory patch identified here and the CTD stabilizes the inactive conformation of Gcn2. Consistent with this, the increased yeast two hybrid interactions between the HisRS and CTD domains evoked by Gcn^-^ substitutions D1327K/D1327A are eliminated under conditions of isoleucine/valine starvation, in which the uncharged cognate tRNAs accumulate and Gcn2 is activated. Furthermore, interaction between the WT HisRS and CTD domains was antagonized in vitro by tRNA, but not by an equal concentration of unstructured mRNA. These findings support the idea that one aspect of Gcn2 activation by uncharged tRNA involves the ability of tRNA bound to the HisRS domain to weaken HisRS/CTD interaction.

**Fig 7:**
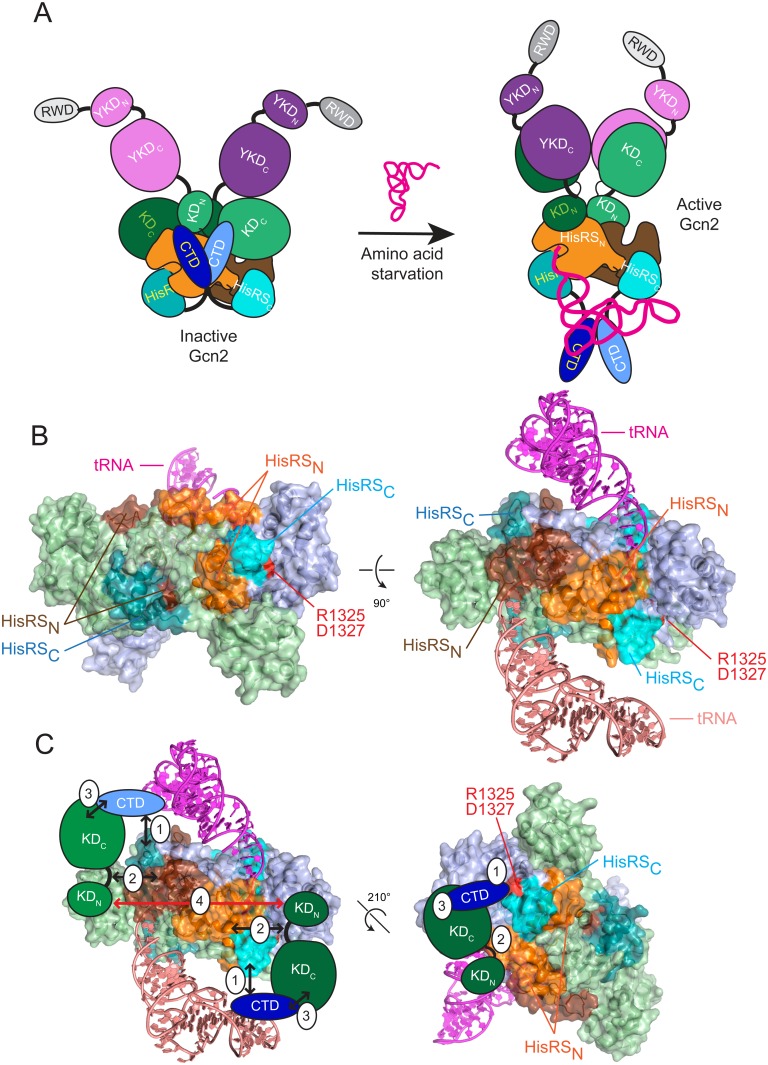
Hypothetical model for the role of the CTD/HisRS domain interaction in stabilizing the inhibitory CTD/KD interaction. (A) (*left*) In nonstarved cells, Gcn2 exists as an inactive dimer with interactions between the CTD (light and dark blue), HisRS domain (light and dark orange for HisRS_N_ and cyan for HisRS_C_) and KD (light and dark green) of each protomer. The KDs assume the anti-parallel mode of dimerization seen in the crystal structure of the inactive KD [12], and the HisRS domains dimerize as observed in crystal structures of authentic HisRS, as shown for *T*. *cruzi* HisRS in panel B. The YKD domain (light and dark purple) is not engaged with the KD owing to the inhibitory KD/CTD interaction, which is stabilized by CTD interaction with the HisRS domain. YKD/CTD interactions might also stabilize this conformation but were omitted for simplicity. (*right*) In amino acid-starved cells, uncharged tRNA binds to the HisRS domains and possibly also the CTD [17] (tRNA binding to only one protomer is depicted for simplicity), evoking a conformational change in the CTD-binding surface of the HisRS domain that triggers dissociation of the CTD/HisRS interaction, which in turn weakens the CTD/KD interaction to allow the stimulatory YKD/KD interaction to prevail. The KDs dimerize in the active back-to-back conformation observed in active PKR dimers [23]. (**B**) Two views of the crystal structure of the *T*. *cruzi* HisRS dimer complexed with HAM (PDB: 1KMM) [51] with modeled uncharged tRNA. HisRS residues Cys-67 to Arg-156, corresponding to the HisRS-N segment of Gcn2 (residues 1028–1120), are colored orange or brown in each protomer; and residues Lys-332 to Iso-380, corresponding to the Gcn2 HisRS-C segment (residues 1315–1383), colored light or dark cyan in each protomer. Arg-341 and Asp-343, corresponding to Gcn2 regulatory residues Arg-1325 and Asp-1327, are colored red. Modeled tRNAs are colored salmon (omitted for clarity in *left* panel) and magenta. (**C**) Two views of the proposed interaction of the Gcn2 CTD with the HisRS-C segment (1) and interaction of the hinge region of the KD with the HisRS-N segment (2), enabling cooperative binding between the KD and CTD (3), which is important for autoinhibition of kinase function in non-starvation conditions. Dimerization of the KDs (4) that occurs in the antiparallel arrangement observed in the crystal structure of the inactive conformation of the Gcn2 KD is indicated by a bidirectional red arrowhead [12].

As noted above, we previously identified an autoinhibitory CTD/KD interaction that appears to be disrupted by tRNA binding to the HisRS domain [17,18]. More recently, we obtained strong evidence that the YKD domain stimulates Gcn2 activity by directly interacting with the KD, and proposed that the inhibitory CTD/KD interaction would compete with this stimulatory YKD/KD interaction, and that tRNA binding to the HisRS domain would shift the balance towards the stimulatory YKD/KD interaction [21]. Integrating our current findings with these previous results suggests the attractive possibility that tRNA binding to the HisRS domain antagonizes the HisRS/CTD interaction to promote a more open conformation of Gcn2 in which the CTD is less tightly bound to the KD. This would allow the YKD to compete more effectively with the CTD for binding to the KD, thereby eliminating autoinhibition by the CTD and correcting structural impediments to kinase activity inherent in the Gcn2 KD (Fig. 7A). Thus, the ability of tRNA binding to weaken the HisRS/CTD interaction would provide a mechanism that serves to replace the inhibitory KD/CTD interaction with the stimulatory YKD/KD interaction. Consistent with the idea that activation of Gcn2 involves rearrangement to a more open conformation lacking domain interactions between the CTD and both the HisRS-C and KD, we found that the activating Gcd^-^ substitution Y1092C/T1328S/A1353V increases the sensitivity of purified WT Gcn2 to digestion by elastase and trypsin. However, high-resolution structural analyses of full-length Gcn2 in the presence and absence of tRNA are clearly required for a rigorous test our model in Fig. 7A.

A distinctive feature of authentic HisRS enzymes is that substrate binding involves an induced-fit mechanism in which histidine binding evokes movement of the “insertion domain” and HisA loop in a way that properly orients a key catalytic arginine residue (Arg-259/Arg-314 of *E*. *coli*/*T*. *cruzi* HisRS) for the formation of histidyl adenylate (HAM). Binding of ATP evokes additional motion of the m2 loop, which moves yet again on ejection of pyrophosphate following HAM formation [40] [41]. The presence of a bound HAM analogue increased the affinity of *E*. *coli* HisRS for tRNA^His^ [42], which might indicate that conformational changes induced by HAM binding also evoke a rearrangement of the active site that optimizes contacts with the acceptor stem of tRNA^His^. Interestingly, it appears that the propensity of HisRS for histidine-induced rearrangement of the active site has been exploited to enable another HisRS-related protein, HisZ, to regulate the catalytic subunit of the octameric subfamily of ATP-phosphoribosyltransferase, HisG, an enzyme of histidine biosynthesis. HisZ contains the allosteric binding site for feedback-inhibition of HisG by histidine, and it is thought that conformational changes in the HisZ pseudo-active site evoked by histidine-binding evoke a remodeling of the HisG dimer interface to stabilize the inactive conformation [43]. Thus, in contrast to Gcn2, the binding of histidine rather than tRNA to the HisRS subunit (HisZ) allosterically regulates the catalytic activity of the binding partner (HisG), and the allosteric molecule (histidine) inhibits rather than stimulates the associated enzyme activity. Nevertheless, it seems plausible to propose that the pseudo-active site in the Gcn2 HisRS domain has evolved to evoke a conformational rearrangement of proximal, surface-exposed residues in response to binding of tRNA (rather than histidine) in the manner envisioned by our model. It is intriguing that the HisA loop, highly conserved among Gcn2 homologs (S2C Fig.), is predicted to be juxtaposed between the 3’ end of tRNA and the HisRS regulatory surface identified here (S5 Fig.), and thus could provide a path for transducing the aminoacylation status of the 3’ end of bound tRNA to the HisRS-CTD regulatory interface.

As noted above, we previously identified a positive regulatory interaction between the HisRS-N segment and the KD and mapped the KD-interacting region between residues 1028–1120 [18], which encompasses the N-terminal dimerization determinant we identified in the Gcn2 HisRS domain [18] (Fig. 7B, see orange and brown surfaces on the two protomers of the *T*. *cruzi* HisRS dimer). Interestingly, this region is contiguous with that corresponding to the portion of the HisRS-C segment that interacts with the CTD [18] (Fig. 7B, light and dark cyan surfaces that harbors the surface-exposed residues altered by the regulatory substitutions D1327A/D1327K and T1328S identified here (red residues in Fig. 7B). It is tempting to propose that the contiguity of the HisRS-N and HisR-C segments will juxtapose their respective interaction partners, the KD and CTD, and enable cooperativity in KD/CTD interaction (Fig. 7B-C). This model also seems compatible with the antiparallel mode of KD dimerization observed in the crystal structure of the inactive state of the Gcn2 KD [12] (Fig. 7C, red arrow connecting KDs in the two protomers). Eliminating the HisRS-C/CTD interaction on tRNA binding, as we proposed above, would eliminate the proposed cooperativity and destabilize CTD binding to the KD, allowing the YKD access to the KD instead (Fig. 7A). Release of the inhibitory HisRS-C/CTD interaction could also facilitate isomerization of the KDs to the parallel mode of dimerization required for their activation, and this alternative mode of dimerization could be further stabilized by the stimulatory YKD-KD interaction (Fig. 7A).

## Materials and Methods

### Sequence and structural alignments

Multiple sequence alignments were generated using MUSCLE at http://www.ebi.ac.uk/Tools/msa/muscle/. ConSurf [44] and PyMOL [45] were used to obtain sequence conservation scores and project the surface representation of sequence conservation on the crystal structure of the *Trypanosoma cruzi* authentic HisRS (PDB:3HRK, Fig. 3E). To obtain a hypothetical model of the Gcn2 HisRS-uncharged tRNA complex, the co-crystal structure of *S*. *cerevisiae* AspRS-tRNA^Asp^ complex (PDB: 1ASZ[Ref: PubMed: 8313877]) was aligned with *T*. *cruzi* HisRS (PDB: 3HRK) by superimposing the highly conserved catalytic core domain (327 residues) using Dali pairwise comparison with default parameters [Ref: PubMed: 19481444]. This alignment produced a robust Z score of 12.9, a RMSD of 3.0 Å, and minimal clashes between tRNA^Asp^ and HisRS. A similar alignment procedure was previously used to model HisRS interaction with tRNA^His^. [Ref: PubMed 7556055; PubMed 11329259] The locations of Gcn2 residues involved in this study were then projected onto the *T*. *cruzi* HisRS crystal structure based on the sequence alignment between Gcn2 and authentic HisRSs (S3 Fig.).

### Plasmids and strains

Plasmids employed are listed in Table 1. For Gcd^-^ mutations identified by random mutagenesis, p2201 was subjected to error-prone PCR mutagenesis using the GeneMorph II kit (Stratagene) by using primer pairs PS-3 (5’-TCTATTTGATAACTCAGTTCCAAC-3’) and PS-4 (5’- TCAGGAATATGTATAAGAAAGGTGAC-3’). The KpnI-NheI 1.8-kb *GCN2* fragment encoding the HisRS-CTD was isolated from plasmid DNA prepared from a pool of *E*. *coli* transformants harboring mutagenized plasmids and subcloned into p2201. Plasmid DNA prepared from a pool of the resulting *E*. *coli* transformants was introduced into yeast strain H1149 and transformants were selected on SC-Ura medium containing 15 mM 3-AT. Resident plasmids were isolated from colony-purified transformants and subjected to DNA sequence analysis to identify the relevant mutations. As multiple mutations generally occurred, QuikChange® site-directed mutagenesis (Stratagene) was used to produce plasmids pSL501, pSL502 and pSL503, containing only single mutations in *GCN2*. Site-directed mutagenesis was also used to generate the novel derivatives (listed in parenthesis) of the following previously constructed plasmids: p2201 (pSL501-pSL507), p722 (pSL508-pSL525), pHQ430 (pSL535-pSL538), pHQ601 (pSL539-pSL541). Plasmids pSL526, pSL527, pSL529, pSL530 and pSL542 were generated by replacing the 3.0-kb BspEI-NheI fragment in pSL101 or pSL102 with the corresponding fragment from p722 derivatives harboring the appropriate *GCN2* mutations.

**Table 1:** Plasmids used in this study.

Name	Description	Source or reference
p722	*CEN6 URA3 GCN2*	[20]
p912	*gcn2-M788V* in p722	[37]
p2201	*gcn2-m2* in p722	Wek, 1995
pSL501	*gcn2- m2/Y1092C* in p2201	This study
pSL502	*gcn2- m2/T1328S* in p2201	This study
pSL503	*gcn2- m2/A1353V* in p2201	This study
pSL507	*gcn2- m2/Y1092C/T1328S/A1353V* in p2201	This study
pSL508	*gcn2-Y1092C* in p722	This study
pSL509	*gcn2-T1328S* in p722	This study
pSL510	*gcn2-A1353V* in p722	This study
pSL511	*gcn2-Y1092C/T1328S/A1353V* in p722	This study
pSL521	*gcn2-R1325A* in p722	This study
pSL522	*gcn2-R1325E* in p722	This study
pSL523	*gcn2-D1327A* in p722	This study
pSL524	*gcn2-D1327K* in p722	This study
pSL525	*gcn2-Y1092C/T1328S/A1353V/D1327K* in p722	This study
pSL101	*P*_*GAL*_-*FLAG-TEV-GCN2*	[36]
pSL102	*P*_*GAL*_-*FLAG-TEV-gcn2-m2* in pSL101	[21]
pSL526	*P*_*GAL*_-*FLAG-TEV-gcn2-Y1092C/T1328S/A1353V* in pSL102	This study
pSL527	*P*_*GAL*_-*FLAG-TEV-gcn2-R1325A* in pSL101	This study
pSL529	*P*_*GAL*_-*FLAG-TEV-gcn2-D1327A* in pSL101	This study
pSL530	*P*_*GAL*_-*FLAG-TEV-gcn2-D1327K* in pSL101	This study
pSL542	*P*_*GAL*_-*FLAG-TEV-gcn2-∆1048–1071* in pSL101	This study
pSH18–34	*lexAop-lacZ* reporter	[52]
pHQ311	*P*_*ADH1*_-*LexA-GCN2(1498–1659)*	[24]
pHQ330	*P*_*GAL*_-*B42*-*GCN2 (1498–1659)*	[24]
pHQ430	*P*_*GAL1*_-*B42*-*GCN2 (970–1497)*	[24]
pSL535	*P*_*GAL1*_-*B42*-*GCN2 (970–1497)-Y1092C/T1328S/A1353V* in pHQ430	This study
pSL536	*P*_*GAL1*_-*B42*-*GCN2 (970–1497)-D1327A* in pHQ430	This study
pSL537	*P*_*GAL1*_-*B42*-*GCN2 (970–1497)-D1327K* in pHQ430	This study
pSL538	*P*_*GAL1*_-*B42*-*GCN2 (970–1497)-Y1092C/T1328S/A1353V/D1327K* in pHQ430	This study
pHQ428	*P*_*GAL1*_-*B42*-*GCN2* (720–999)	[24]
pHQ242	*P*_*ADH1*_-*GST* in 2μ *TRP1* plasmid	[18]
pHQ601	*P*_*ADH1*_-*GST-GCN2(970–1497)* in pHQ242	[18]
pSL539	*P*_*ADH1*_-*gcn2-D1327K* in pHQ601	This study
pSL540	*P*_*ADH1*_-*gcn2-Y1092C/T1328S/A1353V* in pHQ601	This study
pSL541	*P*_*ADH1*_-*gcn2-Y1092C/T1328S/A1353V/D1327K* in pHQ601	This study
pHQ531	*P*_*tac*_-*GST-GCN2(1498–1659)* in pGEX-5x-1	[24]
pHQ551	*P*_*tac*_-*GST-GCN2(568–998)* in pGEX-5x-1	[24]
pHQ541	*P*_*T7*_-*GCN2 (970–1497)* in pGEM-3Z (Promega)	[24]

Yeast strains employed included H1149 (*MATα gcn2∆*::*LEU2 ino1 ura3–52 leu2–3 leu2–112 <HIS4-lacZ>*) [14], HQY132 (*MAT*α *trp1 ura3 his3 lexAop-LEU2 gcn2∆*::*hisG*) [24], and H2684 (*MATa ino1 ura3–52 gcn1Δ gcn2Δ gcn20Δ*) constructed here.

### Protein purification

Transformants of H2684 bearing plasmids pSL101, pSL102, pSL526, pSL527, pSL529, pSL530 and pSL542 were grown to saturation in SC-Ura medium, diluted to A_600_ = 0.2 in SC-Ura containing 10% galactose as carbon source and grown to A_600_ of ∼2.5. Cells were harvested (∼25 g), washed with cold distilled water containing EDTA-free protease inhibitor cocktail (PIC) (Boehringer Mannheim) and 0.5 mM PMSF, resuspended in ice-cold binding buffer (BB) (100 mM sodium phosphate [pH 7.4], 500 mM NaCl, 0.1% Triton X-100, EDTA-free PIC, 1 μg/ml leupeptin, and 1 mM PMSF) and disrupted using SPEX freezer mill (model *6870)*. Lysates were clarified by centrifugation at 39,000 × g for 2 h at 4°C and mixed with 1 ml of M2-FLAG affinity resin (Sigma) overnight at 4°C. The resin was washed three times with 10 vol of BB and Gcn2 was eluted with 100 units of AcTEV protease in 500 μl of 1X TEV buffer (50mM Tris-HCl [pH 8.0], 0.5 mM EDTA, 1mM DTT). The eluates were concentrated with an Amicon Centricon filter (exclusion limit of M_r_ 10,000) and dialyzed against 10 mM Tris-HCl [pH 7.4], 50 mM NaCl, 20% glycerol and stored at −80^0^ C. The eIF2α−ΔC protein was purified from *E*. *coli* as previously described [16]. Preparation of GST and GST fusion proteins of *GCN2* were carried out as described previously [24].

### Reporter assays and Western blot analysis in whole cell extracts (WCEs)

β-galactosidase assays of *HIS4-lacZ* expression were conducted on WCEs prepared from cultures grown in SD medium containing only the required supplements. For non-starvation conditions, saturated cultures were diluted 1:50 and harvested in mid-logarithmic phase after 6 h of growth. For starvation conditions, cultures were grown for 2 h under repressing conditions and then for 6 h after the addition of 3-AT to 10 mM or sulfometuron methyl (SM) to 0.5 μg/ml. β-galactosidase activity was assayed as described previously [46] and expressed as nanomoles of *o*-nitrophenyl-β-D-galactopyranoside hydrolyzed per min per mg of protein.

For Western analysis, WCEs were prepared by trichloroacetic acid extraction, as described previously [47], and immunoblot analysis was conducted as described [24] using phosphospecific antibodies against eIF2α-P (Biosource International) and polyclonal antibodies against eIF2α [48] or Gcn2 [49].

### Kinase and tRNA-binding assays with purified Gcn2

Assays of Gcn2 autophosphorylation were conducted as described previously [11]. Binding of tRNA by Gcn2 was measured using a gel mobility shift assay as described previously [21].

### GST pull-down assays

Pull-downs of LexA-CTD in yeast WCEs with GST-HisRS fusion proteins were conducted as follows. Immobilization of GST fusion proteins on glutathione-Sepharose 4B beads was carried out by incubating the purified fusion proteins at 0.5 μg/μL of beads (bed volume) in buffer A (20mM Tris/HCl pH7.5, 100mM NaCl, 0.2mM EDTA, 1mM DTT) containing 0.1% Triton X-100 at room temperature for 30 min with rocking. The beads were washed and resuspended in the same buffer. Five hundred μg of WCE prepared from pHQ311 transformants of HQY132 was treated with 12,000 units of micrococcal nuclease in the presence of 2mM CaCl_2_ for 10 min at 37°C. Nuclease-treated WCE was then added to beads (10-μL bed volume) containing 5 μg of bound GST fusion proteins and the volume was increased to 200 μL with buffer A. The mixtures were incubated at 4°C for 2 h with rocking. The beads were collected by brief centrifugation in a microcentrifuge, washed three times with 500 μL of buffer A, resuspended in 40 μL of Tris-Glycine SDS Sample Buffer (Novex), and fractionated by SDS-PAGE, transferred to nitrocellulose membranes, and probed with antibodies against GST or LexA. The immune complexes were visualized by enhanced chemiluminescence (ECL; GE Healthcare Life Science) according to the vendor’s instructions.

Pull-downs of [^35^S]-HisRS domain fragments were conducted as follows. *In vitro* transcription/translation with [^35^S]-methionine was conducted using the TNT T7 Coupled Reticulocyte Lysate System (Promega) according to the vendor’s instructions. The resulting [^35^S]-HisRS domain fragments were partially purified by ammonium sulfate precipitation as described previously [50] and resuspended in 50 μL of buffer A (described above) containing 12.5% glycerol. Immobilization of GST fusion proteins on glutathione-Sepharose 4B beads was carried out by incubating the purified fusion proteins at 0.5 μg/μL of beads (bed volume) in buffer A containing 0.1% Triton X-100 at room temperature for 30 min with rocking. The beads were washed and resuspended in the same buffer. Five microliters of [^35^S]-HisRS domain fragments were added to beads (10-μL bed volume) containing 5 μg of bound GST fusion proteins along with the indicated amount of tRNA^Phe^ (Sigma-Aldrich, # R4018), or synthetic mRNA (GGAAUCUCUCUCUCUCUCUCUGCUCUCUCUCUCUCUCUCUCUC) synthesized by T7 polymerase as described in [39], and the volume was increased to 200 μL with buffer A. The mixtures were incubated at 4°C for 2 h with rocking. The beads were collected by brief centrifugation in a microcentrifuge, washed three times with 500 μL of buffer A, resuspended in 40 μL of SDS sample buffer, and fractionated by SDS-PAGE. For detecting the [^35^S]-HisRS domain fragments, the gels were fixed with a solution of isopropanol:water:acetic acid (25:65:10), treated with Amplify (GE Healthcare Life Science), dried, and subjected to fluorography at −80°C.

### Yeast two-hybrid analysis

Plasmids encoding the appropriate LexA- and B42-Gcn2 fusions were cotransformed into yeast strain HQY132. The transformants were selected on synthetic complete medium lacking uracil, histidine, and tryptophan (SC−Ura−His−Trp). Two-hybrid interactions were indicated by β-galactosidase activities in cell extracts of three or more independent transformants. For these assays, cells were grown for 38 h to saturation in SC−Ura−His−Trp and were diluted 1:50 into the same medium containing galactose (2%) and raffinose (1%) as carbon sources (SC/Gal/Raf−Ura−His−Trp). When indicated, sulfometuron was added to the medium at a final concentration of 0.5μg/mL. Cells were harvested in the mid-logarithmic phase after 6 h of growth. β-Galactosidase assays were carried out as described above.

### Protease digestion of Gcn2

Aliquots of 8 μg of purified Gcn2 were incubated with 0.001 units of elastase (Sigma-Aldrich) or 2 pg of trypsin (Sigma-Aldrich) in 10 mM Tris-HCl [pH 7.4], 50 mM NaCl, 20% glycerol for 5 min at room temperature and reactions were quenched by adding SDS sample buffer to a final concentration of 1X followed by heat inactivation at 95°C for 5 min. Digested samples were separated by SDS/PAGE and stained with Coomassie brilliant blue.

## Supporting Information

S1 Fig Combining suppressors *Y1092C*, *T1328S*, and *A1353V* enhances suppression of the *gcn2-m2* mutation. Transformants of *gcn2Δ* strain H1149 with low-copy plasmids containing the indicated *GCN2* alleles were analyzed as described in Fig. 2A except that 3-AT plates were incubated at 37°C. Images were cropped from results obtained from different plates examined in parallel in the same experiment.
(TIF)

S2 Fig Structure-based sequence alignment of the HisRS region of fungal Gcn2 proteins. The multiple sequence alignment of HisRS domains from 42 fungal Gcn2 sequences, identified on the far left by abbreviations of their species of origin, was built using the MUSCLE program. Residues are colored according to evolutionary sequence variation as analyzed with the CONSURF on-line server, with magenta corresponding to the most conserved residues, and dark cyan indicating the most variable. Numbering corresponds to residue positions in full-length *S*. *cerevisiae* Gcn2 (residues 1030–1524). Regions of predicted motifs within the HisRSs are denoted above the *S*. *cerevisiae* sequence, based on the alignment of Gcn2 HisRS sequences with authentic histidyl tRNA-synthetases in S2A-B Fig. Substitutions conferring Gcn^−^ phenotypes are shown in red and those conferring Gcd^−^ phenotypes are shown in green. Residues interacting directly with histidyl adenylate in the *T*. *cruzi* structure are indicated by black letters below the sequence: H/P/S/A signify interaction with the histidyl/phosphate/sugar/adenine moieties respectively. Different portions of the HisRS domain are aligned in panels A to E, encompassing the following residues in full-length *S*. *cerevisiae* Gcn2: **(A)** residues 1030–1125; **(B)** residues 1126–1214; **(C)** residues 1215–1312; **(D)** residues 1313–1386; **(E)** residues 1387–1453; **(F)** residues 1454–1524.
(PDF)

S3 Fig Structure-based sequence alignment of the HisRS region of fungal Gcn2 proteins with authentic histidyl tRNA-synthetases. Multiple sequence alignment of Gcn2 HisRSs from 7 fungal species with 6 different authentic histidyl tRNA-synthetases, was built using the MUSCLE program, Residues are colored according to evolutionary sequence variation as analyzed with the CONSURF on-line server, with magenta corresponding to the most conserved residues, and dark cyan indicating the most variable. Sequences are identified on the far left with abbreviations of their species of origin. Numbering corresponds to residue positions in full-length *S*. *cerevisiae* Gcn2 (residues 1039–1502). Regions of predicted motifs within HisRSs are denoted above the alignment based on their locations in the histidyl tRNA-synthetases. Gcn2 HisRS substitutions examined in this study are shown along the top at their positions in the alignment, with those conferring Gcn^−^ phenotypes shown in red and those conferring Gcd^−^ phenotypes shown in green. Residues interacting directly with histidyl adenylate in the *T*. *cruzi* structure are indicated by black letters below the sequence: H/P/S/A signify interaction with the histidyl/phosphate/sugar/adenine moieties respectively. Different portions of the HisRS domain are aligned in panels A-B, encompassing the following residues in full-length *S*. *cerevisiae* Gcn2: **(A)** residues 1039–1304; **(B)** residues 1305–1502.
(PDF)

S4 Fig HisRS-domain Gcd^-^ substitutions that weaken HisRS/CTD interactions increase protease sensitivity of full-length Gcn2 in vitro. The indicated purified Gcn2 proteins were partially digested with trypsin or elastase and the reaction products were resolved by SDS-PAGE and visualized by Coomassie Blue staining. One microgram of each protein was loaded for the Undigested lanes, whereas 8μg was analyzed for each protease-digested sample. We verified that the major digestion products visible in the protease-treated samples are not visible when higher amounts of the undigested proteins are resolved.
(TIF)

S5 Fig The conserved HisA loop may bridge the pseudo-active site and the regulatory surface in the Gcn2 HisRS domain. (A & B) Two views of the region surrounding the conserved HisA motif (cyan) in the model of the Gcn2 HisRS domain/tRNA complex, colored as in Fig. 3B. Binding of uncharged tRNA (magenta) to the pseudo-active site may remodel the adjacent structure of the HisA loop (highly conserved among Gcn2 homologues, Fig. 3A), which would in turn lead to changes of the regulatory surface (R1325, D1327, T1328, G1338), and ultimately trigger kinase activation.
(TIF)
