# Supplementary figures and images for: Interaction between the tRNA-Binding and C-Terminal Domains of Yeast Gcn2 Regulates Kinase Activity In Vivo
Source: PLoS Genet. 2015 Feb 19;11(2):e1004991. doi: 10.1371/journal.pgen.1004991 (PMC4335047; doi:10.1371/journal.pgen.1004991)

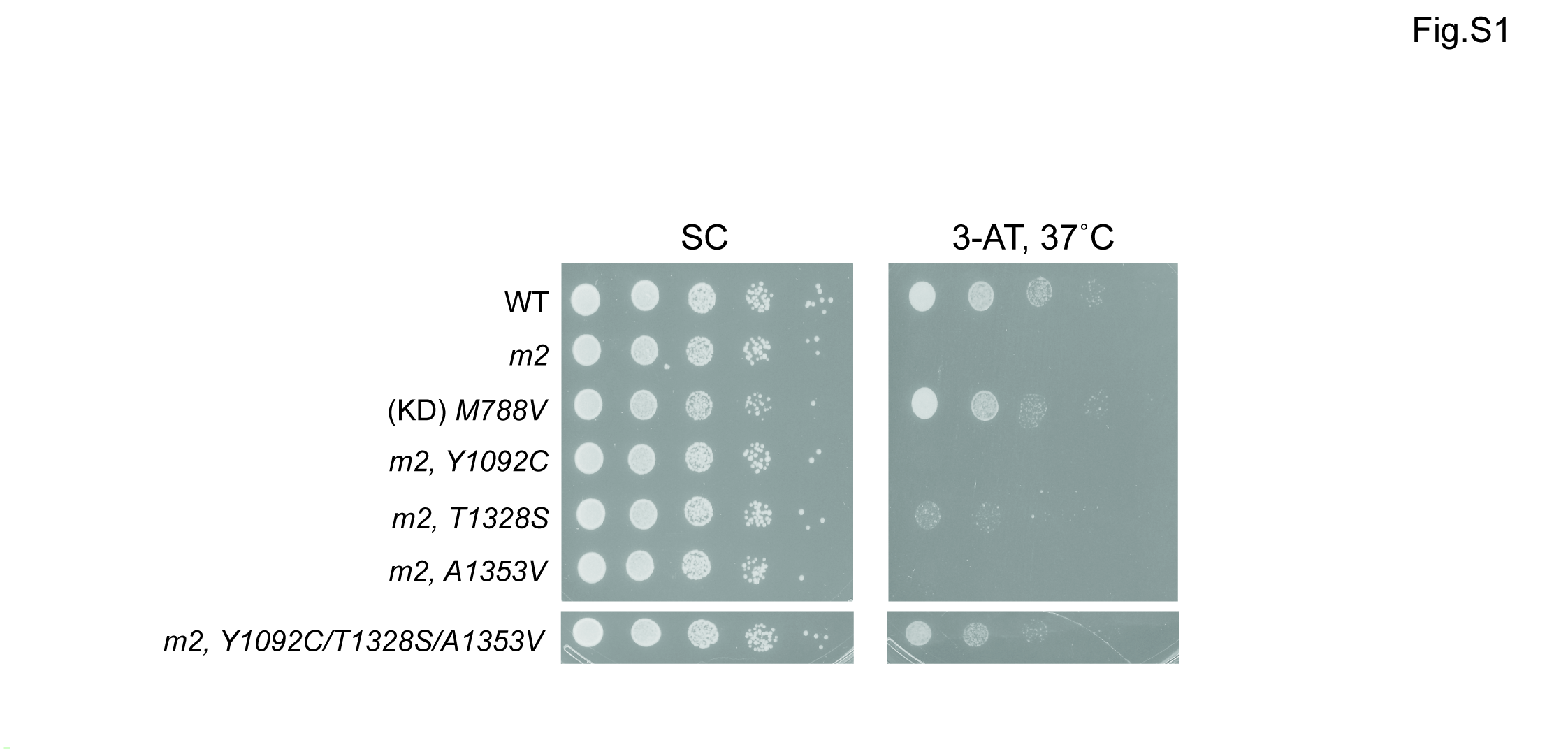

Supplement: S1 Fig — Transformants of gcn2Δ strain H1149 with low-copy plasmids containing the indicated GCN2 alleles were analyzed as described in Fig. 2A except that 3-AT plates were incubated at 37°C. Images were cropped from results obtained from different plates examined in parallel in the same experiment. (TIF) [file pgen.1004991.s001.tif]

Fig.S3-A

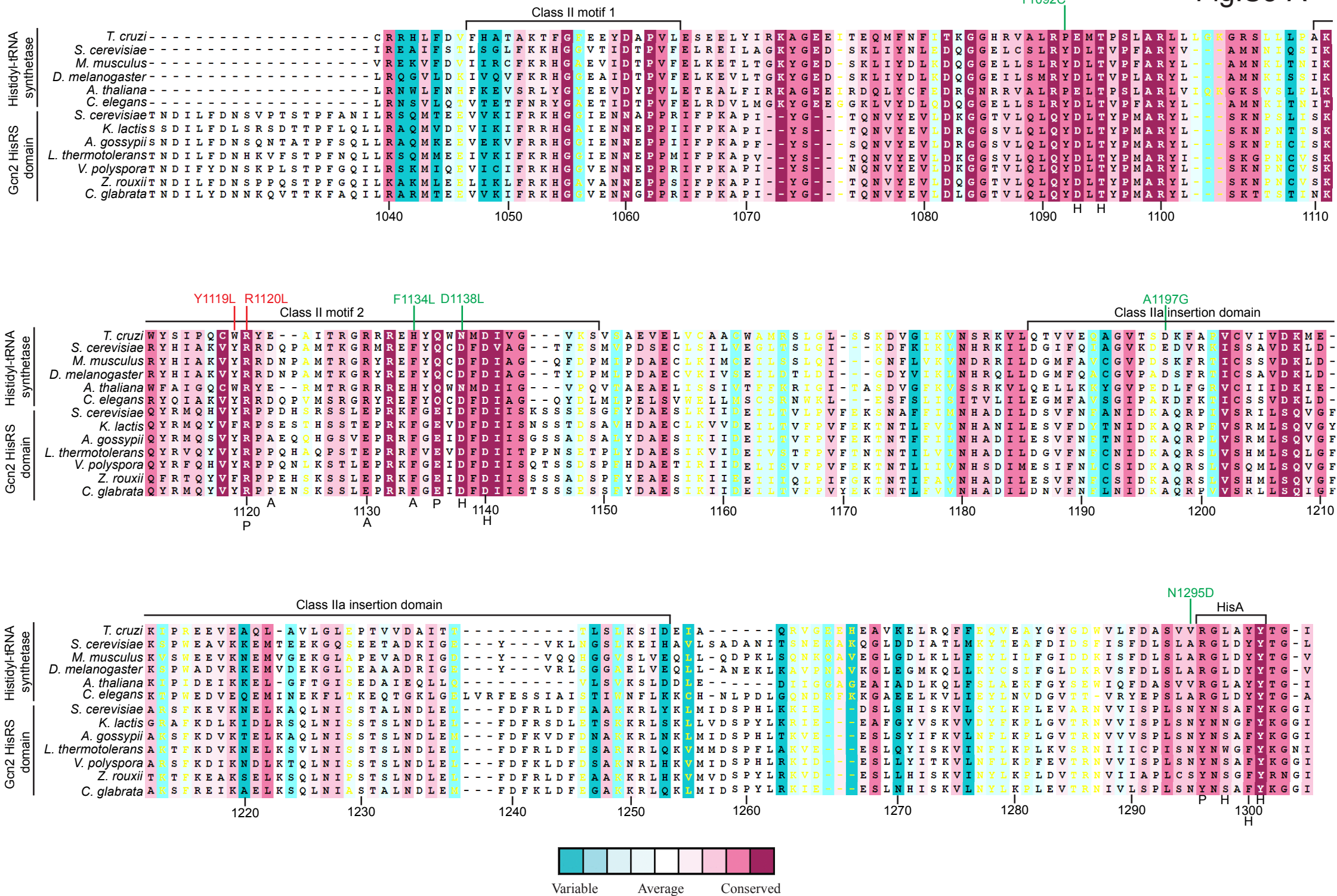

Fig.S3-B

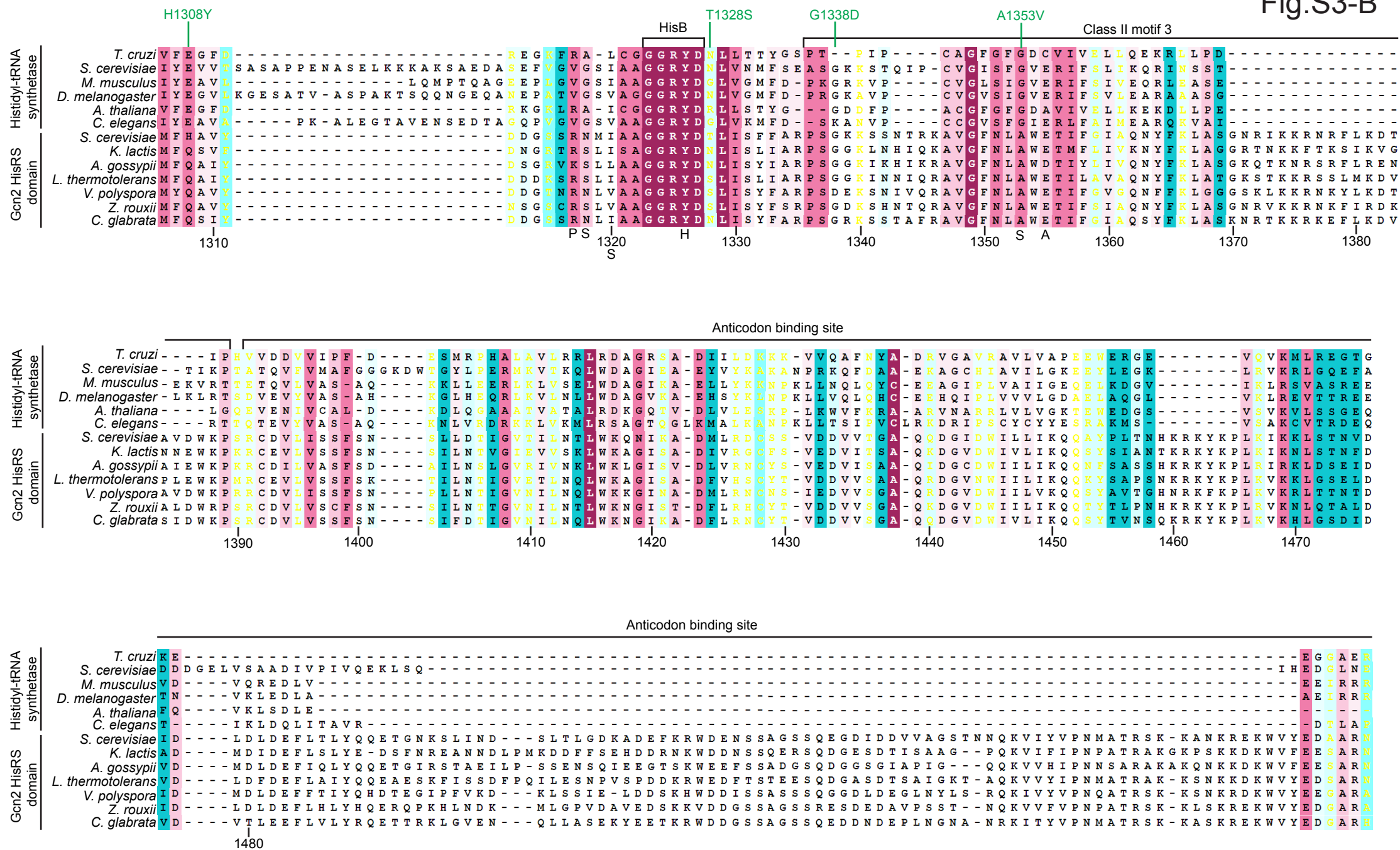

Supplement: S3 Fig — Multiple sequence alignment of Gcn2 HisRSs from 7 fungal species with 6 different authentic histidyl tRNA-synthetases, was built using the MUSCLE program, Residues are colored according to evolutionary sequence variation as analyzed with the CONSURF on-line server, with magenta corresponding to the most conserved residues, and dark cyan indicating the most variable. Sequences are identified on the far left with abbreviations of their species of origin. Numbering corresponds to residue positions in full-length S. cerevisiae Gcn2 (residues 1039–1502). Regions of predicted motifs within HisRSs are denoted above the alignment based on their locations in the histidyl tRNA-synthetases. Gcn2 HisRS substitutions examined in this study are shown along the top at their positions in the alignment, with those conferring Gcn− phenotypes shown in red and those conferring Gcd− phenotypes shown in green. Residues interacting directly with histidyl adenylate in the T. cruzi structure are indicated by black letters below the sequence: H/P/S/A signify interaction with the histidyl/phosphate/sugar/adenine moieties respectively. Different portions of the HisRS domain are aligned in panels A-B, encompassing the following residues in full-length S. cerevisiae Gcn2: (A) residues 1039–1304; (B) residues 1305–1502. (PDF) [file pgen.1004991.s003.pdf]

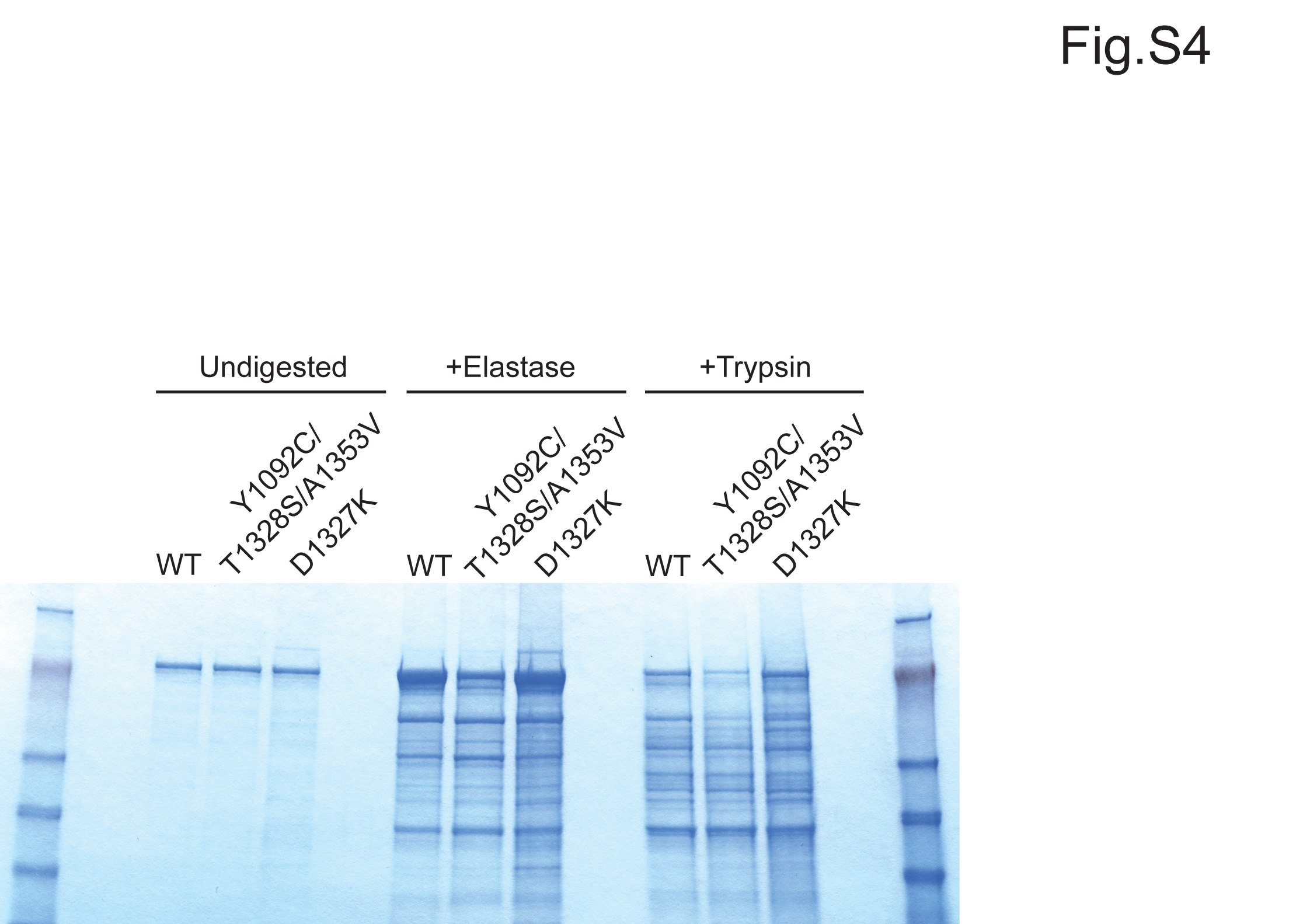

Supplement: S4 Fig — The indicated purified Gcn2 proteins were partially digested with trypsin or elastase and the reaction products were resolved by SDS-PAGE and visualized by Coomassie Blue staining. One microgram of each protein was loaded for the Undigested lanes, whereas 8μg was analyzed for each protease-digested sample. We verified that the major digestion products visible in the protease-treated samples are not visible when higher amounts of the undigested proteins are resolved. (TIF) [file pgen.1004991.s004.tif]

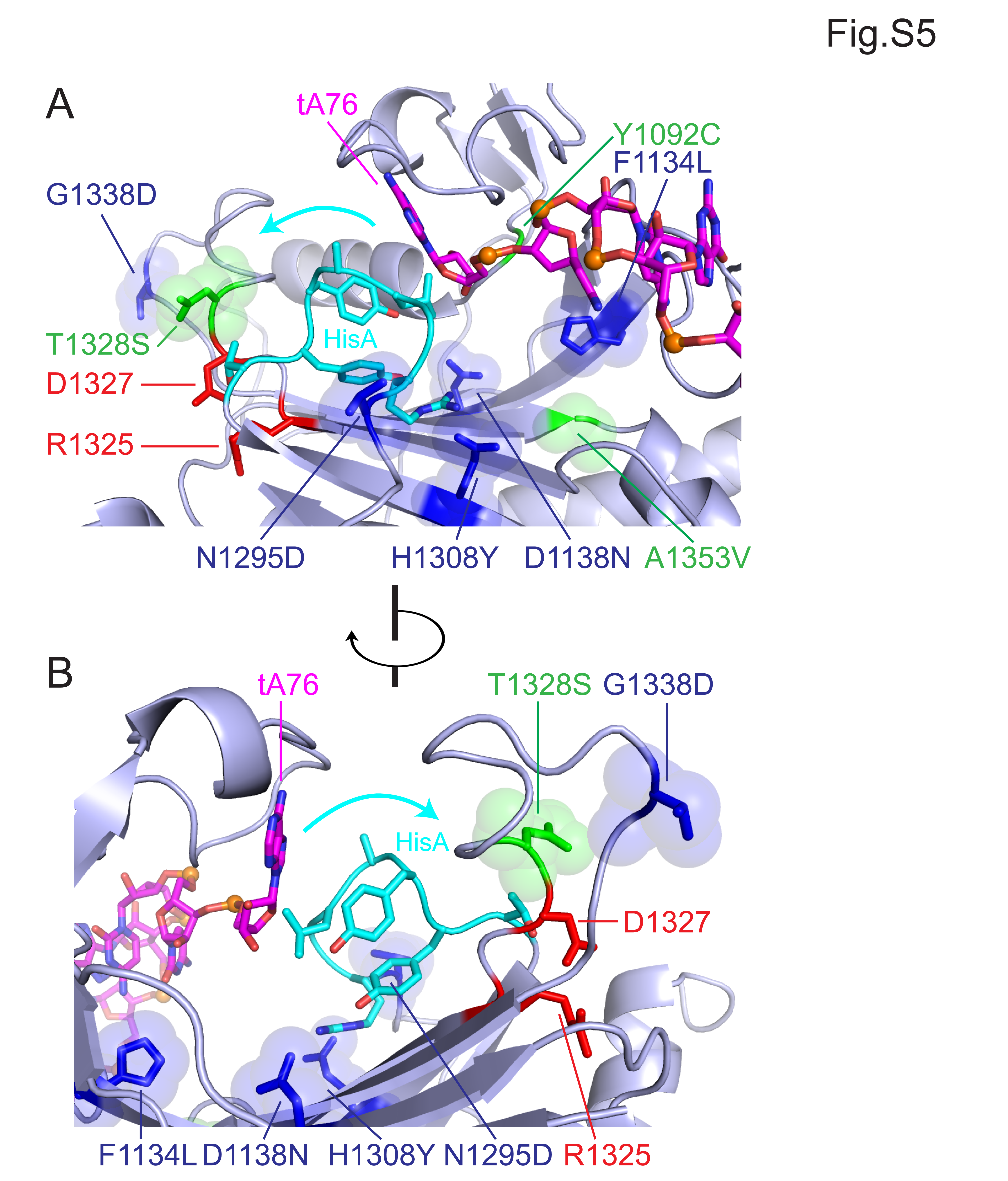

Supplement: S5 Fig — (A & B) Two views of the region surrounding the conserved HisA motif (cyan) in the model of the Gcn2 HisRS domain/tRNA complex, colored as in Fig. 3B. Binding of uncharged tRNA (magenta) to the pseudo-active site may remodel the adjacent structure of the HisA loop (highly conserved among Gcn2 homologues, Fig. 3A), which would in turn lead to changes of the regulatory surface (R1325, D1327, T1328, G1338), and ultimately trigger kinase activation. (TIF) [file pgen.1004991.s005.tif]
