# Supplementary material for: Interaction between the tRNA-Binding and C-Terminal Domains of Yeast Gcn2 Regulates Kinase Activity In Vivo
Source: PLoS Genet. 2015 Feb 19;11(2):e1004991. doi: 10.1371/journal.pgen.1004991 (PMC4335047; doi:10.1371/journal.pgen.1004991)
Supplement: S2 Fig — The multiple sequence alignment of HisRS domains from 42 fungal Gcn2 sequences, identified on the far left by abbreviations of their species of origin, was built using the MUSCLE program. Residues are colored according to evolutionary sequence variation as analyzed with the CONSURF on-line server, with magenta corresponding to the most conserved residues, and dark cyan indicating the most variable. Numbering corresponds to residue positions in full-length S. cerevisiae Gcn2 (residues 1030–1524). Regions of predicted motifs within the HisRSs are denoted above the S. cerevisiae sequence, based on the alignment of Gcn2 HisRS sequences with authentic histidyl tRNA-synthetases in S2A-B Fig. Substitutions conferring Gcn− phenotypes are shown in red and those conferring Gcd− phenotypes are shown in green. Residues interacting directly with histidyl adenylate in the T. cruzi structure are indicated by black letters below the sequence: H/P/S/A signify interaction with the histidyl/phosphate/sugar/adenine moieties respectively. Different portions of the HisRS domain are aligned in panels A to E, encompassing the following residues in full-length S. cerevisiae Gcn2: (A) residues 1030–1125; (B) residues 1126–1214; (C) residues 1215–1312; (D) residues 1313–1386; (E) residues 1387–1453; (F) residues 1454–1524. (PDF) [file pgen.1004991.s002.pdf]

Fig.S2-A

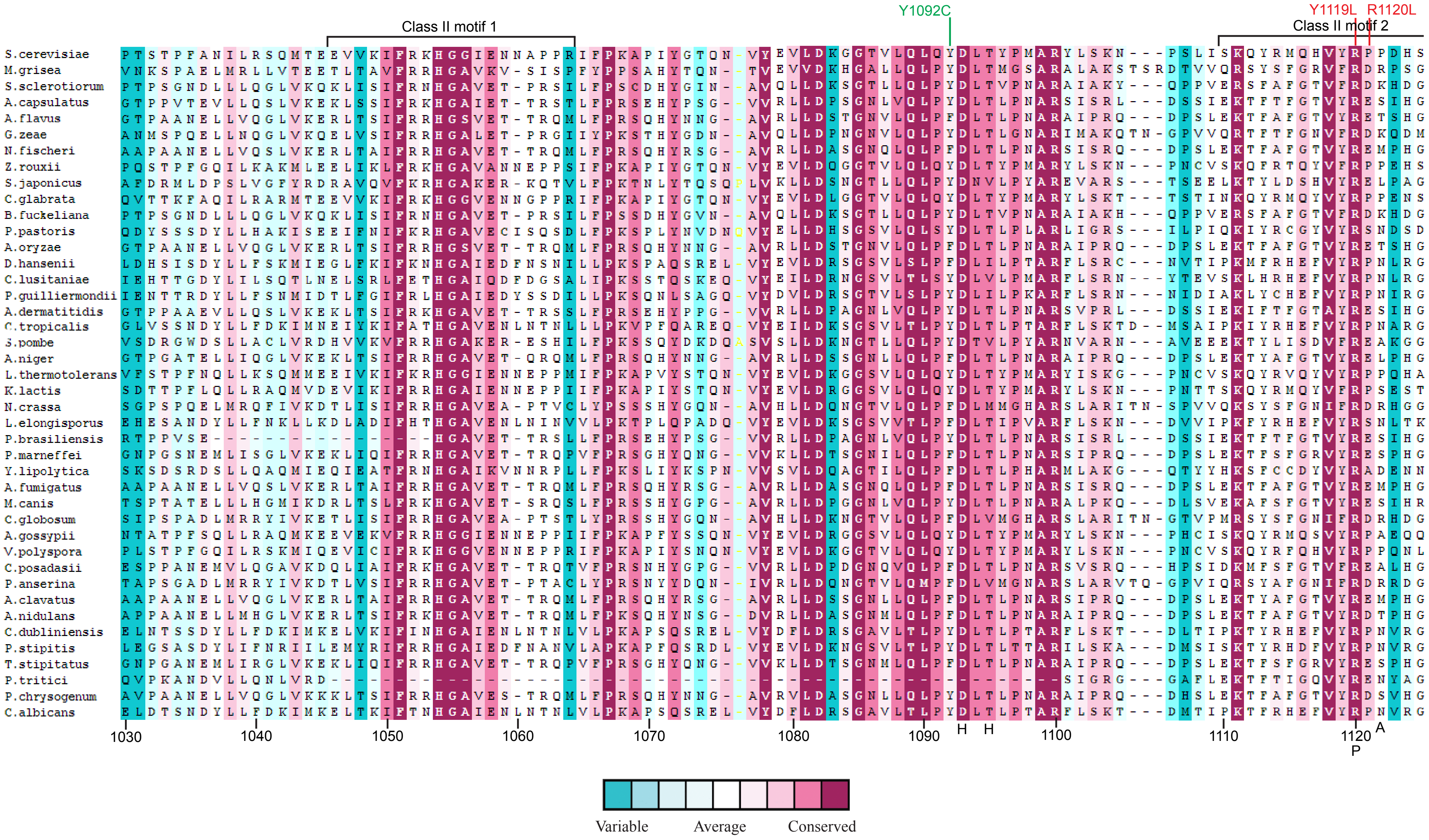
Class II motif 1
Y1092C
Y1119L
R1120L
Class II motif 2
S.cerevisiae
M.grisea
S.sclerotiorum
A.capsulatus
A.flavus
G.zeae
N.fischeri
Z.rouxii
S.japonicus
C.glabrata
B.fuckeliana
P.pastoris
A.oryzae
D.hansenii
C.lusitaniae
P.guilliermondii
A.dermatitidis
C.tropicalis
S.pombe
A.niger
L.thermotolerans
K.lactis
N.crassa
L.elongisporus
P.brasiliensis
P.marneffei
Y.lipolytica
A.fumigatus
M.canis
C.globosum
A.gossypii
V.polyspora
C.posadasii
P.anserina
A.clavatus
A.nidulans
C.dubliniensis
P.stipitis
T.stipitatus
P.tritici
P.chrysogenum
C.albicans
1030
1040
1050
1060
1070
1080
1090
H
H
1100
1110
1120
A
P
Variable
Average
Conserved

Fig.S2-B

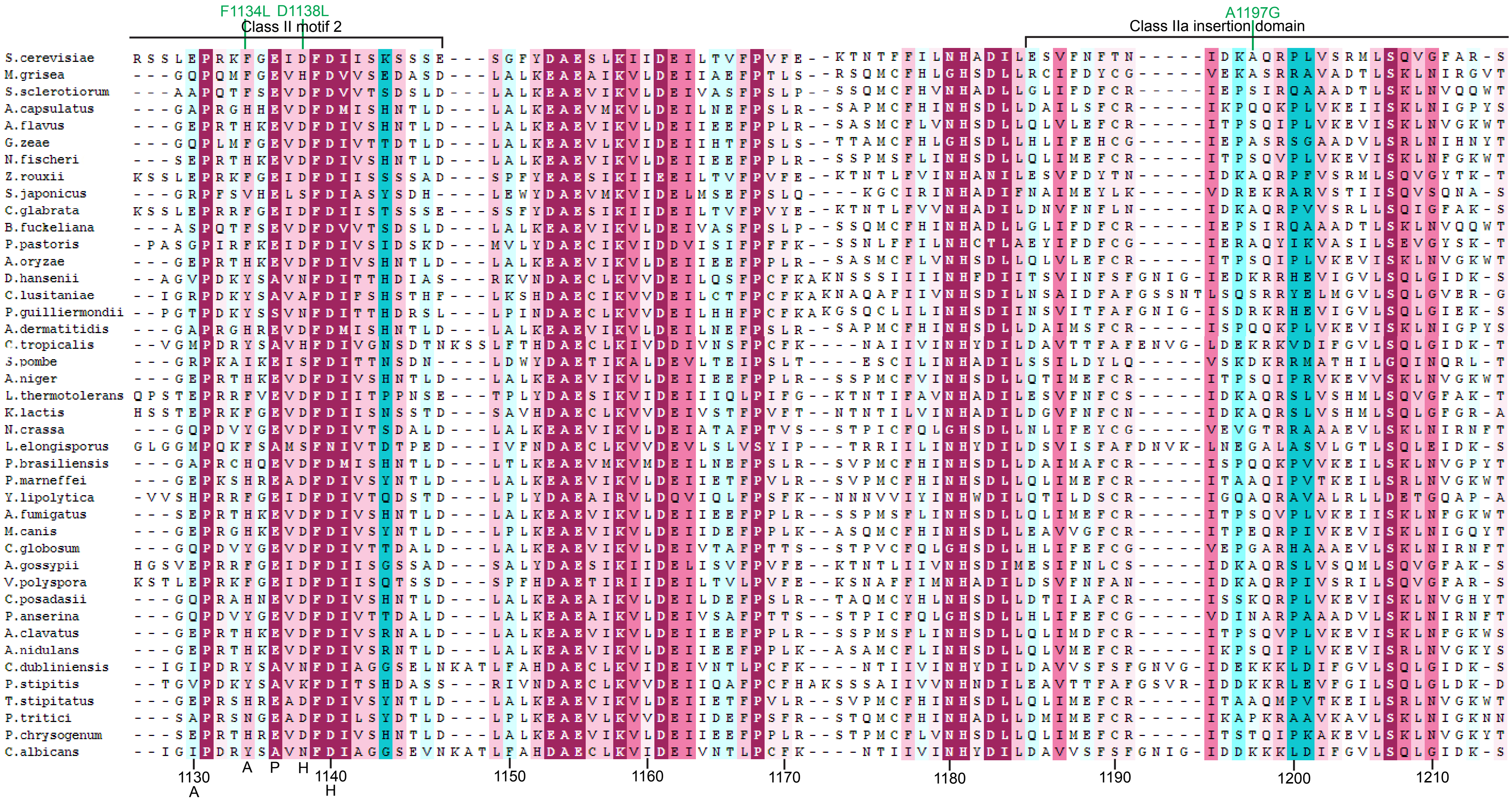
F1134L D1138L
Class II motif 2
A1197G
Class IIa insertion domain
S.cerevisiae
M.grisea
S.sclerotiorum
A.capsulatus
A.flavus
G.zeae
N.fischeri
Z.rouxii
S.japonicus
C.glabrata
B.fuckeliana
P.pastoris
A.oryzae
D.hansenii
C.lusitaniae
P.guilliermondii
A.dermatitidis
C.tropicalis
S.pombe
A.niger
L.thermotolerans
K.lactis
N.crassa
L.elongisporus
P.brasiliensis
P.marneffei
Y.lipolytica
A.fumigatus
M.canis
C.globosum
A.gossypii
V.polyspora
C.posadasii
P.anserina
A.clavatus
A.nidulans
C.dubliniensis
P.stipitis
T.stipitatus
P.tritici
P.chrysogenum
C.albicans
1130
A
A
P
H
1140
H
1150
1160
1170
1180
1190
1200
1210

Fig.S2-C

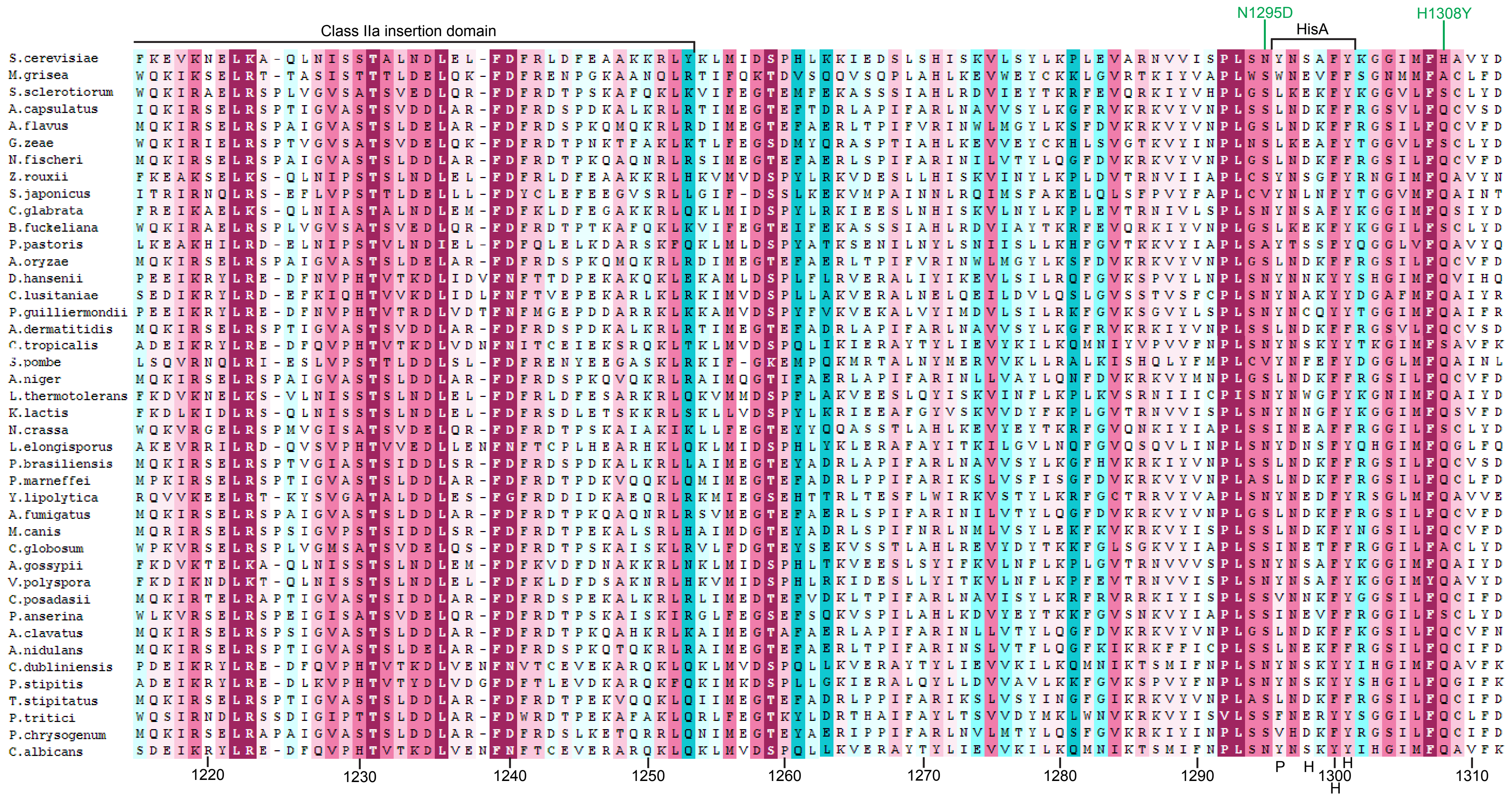
Class IIa insertion domain
N1295D
H1308Y
HisA
P
H
H
H
1220
1230
1240
1250
1260
1270
1280
1290
1300
1310

Fig.S2-D

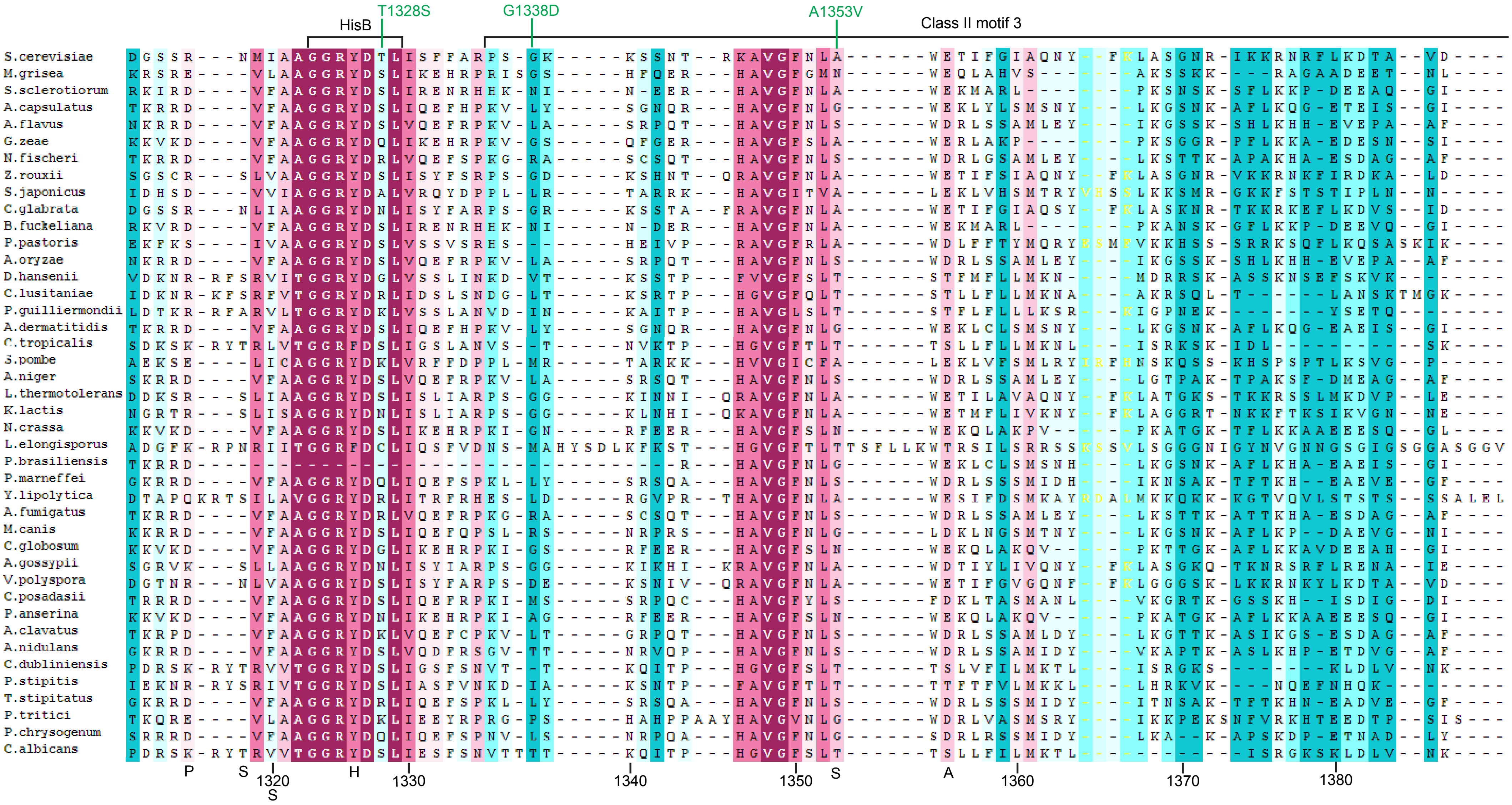
HisB
T1328S
G1338D
A1353V
Class II motif 3
S.cerevisiae
M.grisea
S.sclerotiorum
A.capsulatus
A.flavus
G.zeae
N.fischeri
Z.rouxii
S.japonicus
C.glabrata
B.fuckeliana
P.pastoris
A.oryzae
D.hansenii
C.lusitaniae
P.guilliermondii
A.dermatitidis
C.tropicalis
S.pombe
A.niger
L.thermotolerans
K.lactis
N.crassa
L.elongisporus
P.brasiliensis
P.marneffei
Y.lipolytica
A.fumigatus
M.canis
C.globosum
A.gossypii
V.polyspora
C.posadasii
P.anserina
A.clavatus
A.nidulans
C.dubliniensis
P.stipitis
T.stipitatus
P.tritici
P.chrysogenum
C.albicans
P
S
1320
S
H
1330
1340
1350
S
A
1360
1370
1380

Fig.S2-E

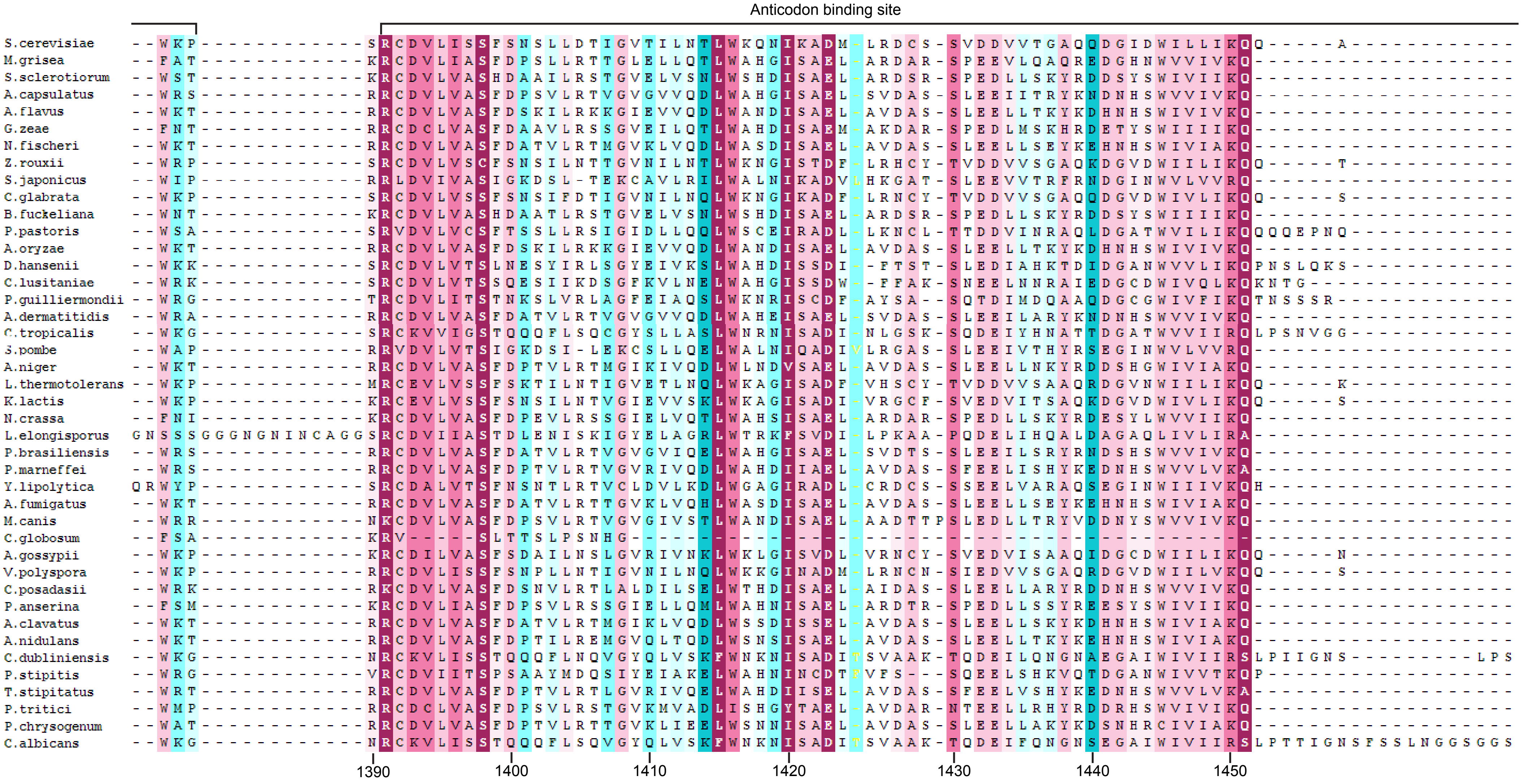
Anticodon binding site
S.cerevisiae
M.grisea
S.sclerotiorum
A.capsulatus
A.flavus
G.zeae
N.fischeri
Z.rouxii
S.japonicus
C.glabrata
B.fuckeliana
P.pastoris
A.oryzae
D.hansenii
C.lusitaniae
P.guilliermondii
A.dermatitidis
C.tropicalis
S.pombe
A.niger
L.thermotolerans
K.lactis
N.crassa
L.elongisporus
P.brasiliensis
P.marneffei
Y.lipolytica
A.fumigatus
M.canis
C.globosum
A.gossypii
V.polyspora
C.posadasii
P.anserina
A.clavatus
A.nidulans
C.dubliniensis
P.stipitis
T.stipitatus
P.tritici
P.chrysogenum
C.albicans
1390
1400
1410
1420
1430
1440
1450

Fig.S2-F

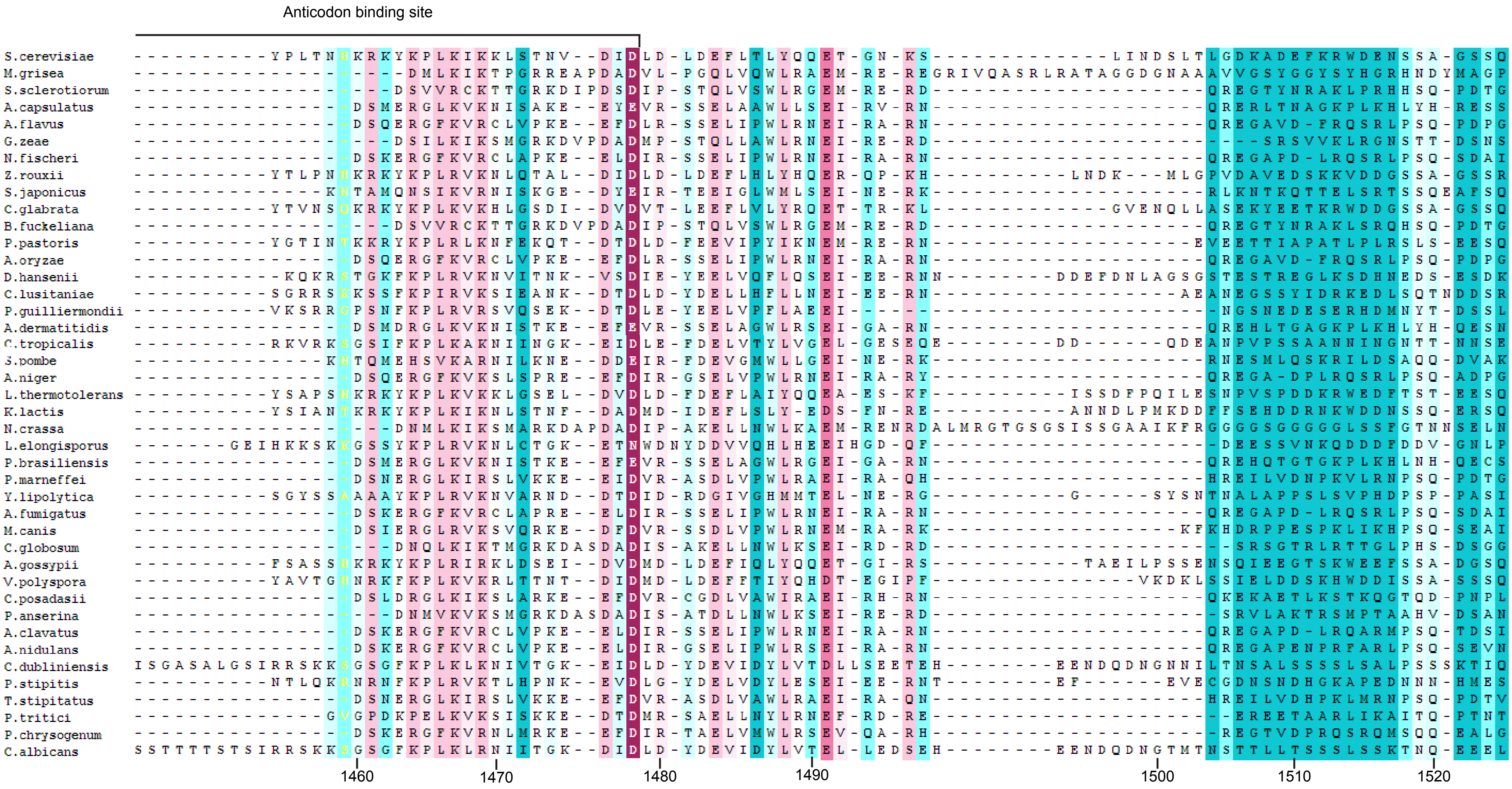
Anticodon binding site
S.cerevisiae
M.grisea
S.sclerotiorum
A.capsulatus
A.flavus
G.zeae
N.fischeri
Z.rouxii
S.japonicus
C.glabrata
B.fuckeliana
P.pastoris
A.oryzae
D.hansenii
C.lusitaniae
P.guilliermondii
A.dermatitidis
C.tropicalis
S.pombe
A.niger
L.thermotolerans
K.lactis
N.crassa
L.elongisporus
P.brasiliensis
P.marneffei
Y.lipolytica
A.fumigatus
M.canis
C.globosum
A.gossypii
V.polyspora
C.posadasii
P.anserina
A.clavatus
A.nidulans
C.dubliniensis
P.stipitis
T.stipitatus
P.tritici
P.chrysogenum
C.albicans
1460
1470
1480
1490
1500
1510
1520
